# Systemic Melanoma Therapy in Patients with Pre-Existing Heart Failure: An HF-Centered Decision Framework and Critical Narrative Review

**DOI:** 10.3390/jcm15187022

**Published:** 2026-09-10

**Authors:** Daniela-Vasilica Serban, Diana-Maria Mateescu, Daniela Crainic, Nina Ivanovic, Roxana Manuela Fericean, Florina Maria Bojin, Ana-Olivia Toma, Elena Daniela Jurj, Emilia Clej, Virgil Paunescu

**Affiliations:** 1 Doctoral School, “Victor Babeș” University of Medicine and Pharmacy Timișoara, 300041 Timișoara, Romania; daniela.serban@umft.ro (D.-V.S.); daniela.crainic@umft.ro (D.C.); nina.ivanovic@umft.ro (N.I.); daniela.adam@umft.ro (E.D.J.); emilia.clej@umft.ro (E.C.); 2 Center for the Morphologic Study of the Skin (MORPHODERM), “Victor Babeș” University of Medicine and Pharmacy Timișoara, 300041 Timișoara, Romania; manuela.fericean@umft.ro; 3 Department of Infectious Diseases, Clinical Hospital CF Timișoara, 1 Gării Street, 300166 Timișoara, Romania; diana.mateescu@umft.ro; 4 Center of Immuno-Physiology and Biotechnologies, Department of Functional Sciences, “Victor Babeș” University of Medicine and Pharmacy Timișoara, 300041 Timișoara, Romania; vpaunescu@umft.ro; 5 Research Center for Gene and Cellular Therapies in the Treatment of Cancer—OncoGen, Timis County Emergency Clinical Hospital “Pius Brinzeu”, 300723 Timișoara, Romania; 6 Center for Drug Data Analysis, Cheminformatics, and the Internet of Medical Things, “Victor Babeș” University of Medicine and Pharmacy Timișoara, Eftimie Murgu Square, No. 2, 300041 Timișoara, Romania

**Keywords:** melanoma, pre-existing heart failure, cardio-oncology, BRAF inhibitors, MEK inhibitors, immune checkpoint inhibitors, cancer therapy-related cardiac dysfunction, myocarditis, drug–drug interactions, multidisciplinary care

## Abstract

**Background/Objectives**: General cardio-oncology guidance describes cardiovascular toxicity from BRAF/MEK inhibitors and immune checkpoint inhibitors (ICIs), but it does not resolve how treatment decisions should change when heart failure (HF) is already present. This critical narrative review separates direct HF-specific evidence from indirect evidence and proposes an HF-centered framework for systemic melanoma. **Methods**: MEDLINE/PubMed, Embase, and the Cochrane Library were searched for English-language sources published from January 2015 to April 2026, with targeted guideline, prescribing-information, interaction, and geriatric-oncology updates through July 2026. Sources were classified as direct, indirect, or extrapolated. This review follows SANRA principles but does not claim systematic-review conduct, pooled estimates, formal risk-of-bias assessment, or GRADE certainty. **Results**: Only case-level evidence directly describes systemic melanoma treatment in established HF; most cardiotoxicity rates are derived from selected longitudinal and real-world cohorts. We therefore organize decisions across four domains: HF phenotype and current stability; oncological urgency and alternatives; treatment-specific toxicity phenotype; and detectability, reversibility, and patient priorities. Each domain is graded ordinally and generates its own decision output, and explicit precedence rules resolve situations in which several domains are simultaneously abnormal; the pathway from assessment to documented output is presented as a decision flowchart and applied to illustrative clinical cases. This approach modifies interpretation of symptoms, biomarkers, ventricular function, surveillance, drug interactions, treatment interruption, and rechallenge. Every actionable statement is labeled as guideline-supported or author-proposed, therapy-specific baseline and follow-up monitoring are tabulated separately for each treatment class, and heart failure with preserved ejection fraction is addressed as a phenotype in which clinical deterioration may occur without any change in ejection fraction. Patient-level priorities in older adults—survival, quality of life, avoidance of hospitalization, tolerance of frequent monitoring, oral versus infusion treatment, and functional expectation—are specified as items to be recorded rather than inferred. **Conclusions**: Stable HF is not an automatic contraindication to effective melanoma therapy, whereas recent or active decompensation requires stabilization or monitored treatment when oncological delay is unsafe. The proposed framework operationalizes guideline principles for an understudied population while making the limits of the evidence explicit. It makes a procedural rather than an empirical claim: it is a transparent structure for documenting a decision that must be made anyway, not an algorithm derived from or validated against outcome data.

## 1. Introduction

Melanoma is the most lethal form of skin cancer, accounting for the vast majority of skin cancer-related deaths despite representing only a minority of cases by incidence. Historically, the prognosis of metastatic melanoma was dismal, with five-year survival rates of 15–20% in the era preceding modern immunotherapy. The discovery of activating somatic mutations in the BRAF oncogene—present in approximately 40–50% of cutaneous melanomas, predominantly at codon V600—and the elucidation of T-cell immune checkpoint pathways revolutionized therapeutic approaches and fundamentally altered the natural history of this disease.

An estimated 34% of patients with unresectable or metastatic melanoma harboring a BRAF V600E or V600K mutation were alive and 19% remained progression-free at five years after first-line treatment with the combination of dabrafenib and trametinib, with survival curves appearing to plateau between years three and five [1]. In the immunotherapy realm, patients harboring a BRAF mutation and receiving first-line immune checkpoint blockade with ipilimumab plus nivolumab demonstrated a five-year overall survival rate of 60%, raising the question of whether a true cure may be achievable for a subset of patients [2].

This improved longevity introduces a new clinical paradigm: patients with advanced melanoma increasingly survive long enough to develop, or present with, comorbidities that were previously of limited oncological relevance. HF—affecting approximately 1–2% of the general population and more than 10% of individuals older than 70 years—is one such comorbidity. Advanced melanoma also disproportionately affects older adults, among whom frailty, multimorbidity, polypharmacy, impaired renal reserve, and functional limitations may coexist with HF. Chronological age, frailty, and HF are related but not interchangeable constructs, and each may modify treatment feasibility and monitoring needs.

The two principal systemic treatment classes considered in this review—BRAF/MEK inhibitors and immune checkpoint inhibitors—have distinct mechanisms and cardiovascular toxicity profiles. BRAF/MEK inhibitors are molecularly targeted small-molecule therapies, whereas ICIs are immunotherapies; they should not be grouped under the imprecise label “targeted biological therapies.” Their cardiovascular effects range from asymptomatic reductions in left ventricular function to fulminant immune-mediated myocarditis. Patients with pre-existing HF may have reduced cardiac reserve, but the magnitude of any additional treatment-related risk is uncertain because clinically significant cardiac disease, advanced age, and frailty have commonly been excluded or incompletely characterized in pivotal studies [3,4].

This critical narrative review asks a narrower question than general cardio-oncology reviews: what changes when HF precedes melanoma therapy rather than developing as a treatment complication? It does not generate new evidence or pooled risk estimates. It distinguishes findings obtained directly in patients with established HF from findings in unselected melanoma cohorts and recommendations extrapolated from broader cardio-oncology populations. Its distinctive contribution is an HF-centered four-domain decision framework that integrates HF substrate and stability, oncological urgency, treatment-specific toxicity phenotype, and detectability/reversibility together with patient priorities.

It is reasonable to ask how a review that insists on the weakness of the evidence can, in the same breath, propose a structured framework, and we prefer to state the relationship between the two at the outset rather than leave it to be inferred. The framework does not derive recommendations from the evidence, does not convert case-level observations into thresholds, and does not reduce the uncertainty that this review describes; none of it is validated. What it does is organize a decision that clinicians are obliged to make regardless of how thin the evidence is, and make the components of that decision visible: which variable was weighed against which, what trade-off was accepted, and what would reverse it. The claim is therefore procedural rather than empirical, and two consequences follow that are applied throughout this article. Every actionable statement is labeled as guideline-supported or author-proposed (Section 2.5), and the framework is presented as a documentation structure rather than as a decision algorithm, a risk score, or a substitute for multidisciplinary judgement (Section 4.3).

## 2. Materials and Methods

### 2.1. Search Strategy and Eligibility Criteria

This critical narrative review was developed according to the Scale for the Assessment of Narrative Review Articles (SANRA) [5]. MEDLINE/PubMed, Embase, and the Cochrane Library were searched for publications from January 2015 through April 2026. Search concepts combined melanoma and systemic therapy terms with HF, cardiotoxicity, myocarditis, ventricular dysfunction, and cardio-oncology terms. A targeted update of guidelines, official prescribing information, interaction resources, and geriatric-oncology literature was completed in July 2026. The database-adapted search syntax is provided in Appendix A.

Boolean operators and database-specific controlled vocabulary were used. Reference lists of eligible articles and major guidelines were hand-searched, and official product information and a recognized oncology interaction database were consulted for drug-specific interaction statements. Conference abstracts, preprints, and non-peer-reviewed clinical claims were excluded. PRISMA 2020 is a reporting guideline for systematic reviews [6]; the present article remains a critical narrative review and does not claim duplicate systematic screening, protocol registration, or exhaustive study capture. Because the original search was not prospectively exported with a complete audit trail, exact initial hit and exclusion counts cannot be reconstructed without false precision, and no retrospective numerical flow was fabricated.

### 2.2. Inclusion and Exclusion Criteria

Eligible sources included randomized trials, prospective and retrospective cohorts, pharmacovigilance studies, case series, systematic reviews, consensus statements, international guidelines, and official prescribing information. Case reports were included only when they provided clinically distinctive information relevant to established HF, diagnostic ambiguity, reversibility, or treatment decisions. Studies with inadequately defined cardiovascular outcomes were not used to support quantitative estimates. English-language publications were included.

### 2.3. Data Extraction and Synthesis

We extracted study design, population, baseline cardiovascular status, age and geriatric characteristics when reported, melanoma regimen, event definition, incidence, timing, diagnostic approach, surveillance strategy, treatment, reversibility, and outcomes. Because definitions and ascertainment methods differed substantially, no meta-analysis was performed. Evidence was classified as direct when it involved patients with established HF, indirect when derived from unselected melanoma cohorts without a dedicated HF analysis, and extrapolated when derived from other cancer populations, general HF trials, product labels, or expert consensus. This hierarchy was used to calibrate the wording of clinical recommendations. The illustrative clinical vignettes used in Section 3.7 and in the Discussion to demonstrate the framework are author-constructed composites derived from published case-level reports, the cohort observations tabulated in this review, guideline recommendations and prescribing information; they do not describe identifiable individual patients and no clinical records were accessed.

### 2.4. Evidence Applicability and Qualitative Appraisal

Direct clinical evidence in melanoma patients with established HF is sparse. Table 1 therefore identifies whether each major evidence stream is direct, indirect, or extrapolated and prevents incidence estimates from unselected cohorts from being interpreted as HF-specific rates. Study design, directness, consistency, event definition, surveillance intensity, and applicability to older or frail patients were considered qualitatively.

Because this is a narrative review, duplicate independent screening, a formal study-level risk-of-bias tool, and GRADE certainty ratings were not applied. To improve transparency, the final corpus is explicitly accounted for: 50 core sources comprised 28 primary clinical, pharmacovigilance, or case-based studies; 14 peer-reviewed reviews, guidelines, consensus statements, or professional recommendations; seven official product labels; and one curated oncology interaction database. This review can integrate existing knowledge, expose inconsistencies, and identify research gaps, but it cannot establish pooled incidence, comparative safety, or causal treatment effects.

### 2.5. Labeling of Guideline-Supported and Author-Proposed Statements

Because the population addressed here is one for which direct evidence is case-level, there is a real risk that a structured expert synthesis will be read as guidance. To keep the boundary between evidence and expert judgement visible at the point of use rather than only in the limitations, every actionable statement in this review carries one of two labels. A statement is designated guideline-supported (G) when it reproduces a recommendation, definition, or explicit statement contained in the 2022 ESC cardio-oncology guidelines, the Heart Failure Association/International Cardio-Oncology Society baseline risk position statement, the IC-OS consensus definitions, current heart failure guidelines, or approved product information [3,22,23,24,25,26,30,31,32,33,34,35]. A statement is designated author-proposed (AP) when it is a risk-adapted extrapolation constructed by the authors for the specific situation of pre-existing HF and is not stated in any of those sources.

The author-proposed category includes, but is not limited to, the four-domain framework and its ordinal descriptors, the precedence rules and composite patterns, the phenotype-specific proposals for HFrEF, HFpEF, predominant right ventricular failure, device dependence and advanced HF, the earlier imaging reassessment proposed for unstable or very-high-risk patients, the biomarker and imaging thresholds referenced to the patient’s own baseline, and the interruption, resumption and rechallenge criteria proposed for patients whose baseline is already abnormal. These labels are applied in Section 3.1.3, Section 3.1.4, Section 3.5.2, Section 3.6.1, and Section 3.6.2. No statement labeled author-proposed should be cited as a guideline recommendation, used as a validated threshold, or treated as a standard of care.

### 2.6. Terminological Conventions

Two conventions are applied throughout this text, the tables and the figures. First, the combinations of a BRAF inhibitor with a MEK inhibitor that are approved for melanoma (dabrafenib–trametinib, encorafenib–binimetinib, vemurafenib–cobimetinib) are consistently called BRAF/MEK inhibitors. The term “RAF/MEK inhibitors” is used only where the exact title of the corresponding HFA-ICOS baseline risk proforma is being cited, and in those instances the proforma is named explicitly so that the reader can see that the two terms refer to the same regimens [26]. Second, BRAF/MEK inhibitors are described as molecularly targeted small-molecule therapies and checkpoint inhibitors as immunotherapies; the two classes are never grouped under a single label such as “targeted biological therapies”, because their toxicity phenotypes, time course and management differ.

## 3. Results

### 3.1. Pre-Existing Heart Failure as the Primary Clinical Substrate

#### 3.1.1. Why Pre-Existing HF Changes Risk Interpretation

Established HF should not be treated as a binary comorbidity. Clinical vulnerability differs across HFrEF, HFmrEF, HFpEF, and HF with improved ejection fraction and is further modified by current congestion, recent hospitalization, right ventricular dysfunction, pulmonary hypertension, valvular disease, arrhythmia burden, device dependence, renal dysfunction, blood pressure, and tolerance of guideline-directed therapy. A stable ambulatory patient with recovered LVEF is not equivalent to a patient with active decompensation or advanced HF. Because significant cardiovascular disease was commonly excluded or incompletely characterized in pivotal melanoma trials, the incremental treatment-related risk attributable to established HF remains unknown [3,26,36].

Pre-existing HF also reduces the specificity of conventional toxicity signals. Dyspnea, fatigue, edema, elevated BNP/NT-proBNP, low-level troponin elevation, and reduced LVEF may predate cancer therapy. New toxicity should therefore be defined by change from a documented patient-specific baseline and interpreted alongside congestion, ECG findings, serial biomarker dynamics, GLS/LVEF trajectory, treatment timing, and competing causes such as infection, anemia, pulmonary embolism, acute coronary syndrome, malignant effusion, renal deterioration, or tumor progression. This principle is particularly important for ICI myocarditis, in which a normal LVEF does not exclude disease and chronic biomarker abnormalities can complicate diagnostic thresholds.

#### 3.1.2. Four-Domain HF-Centered Decision Framework

**Figure 1:**
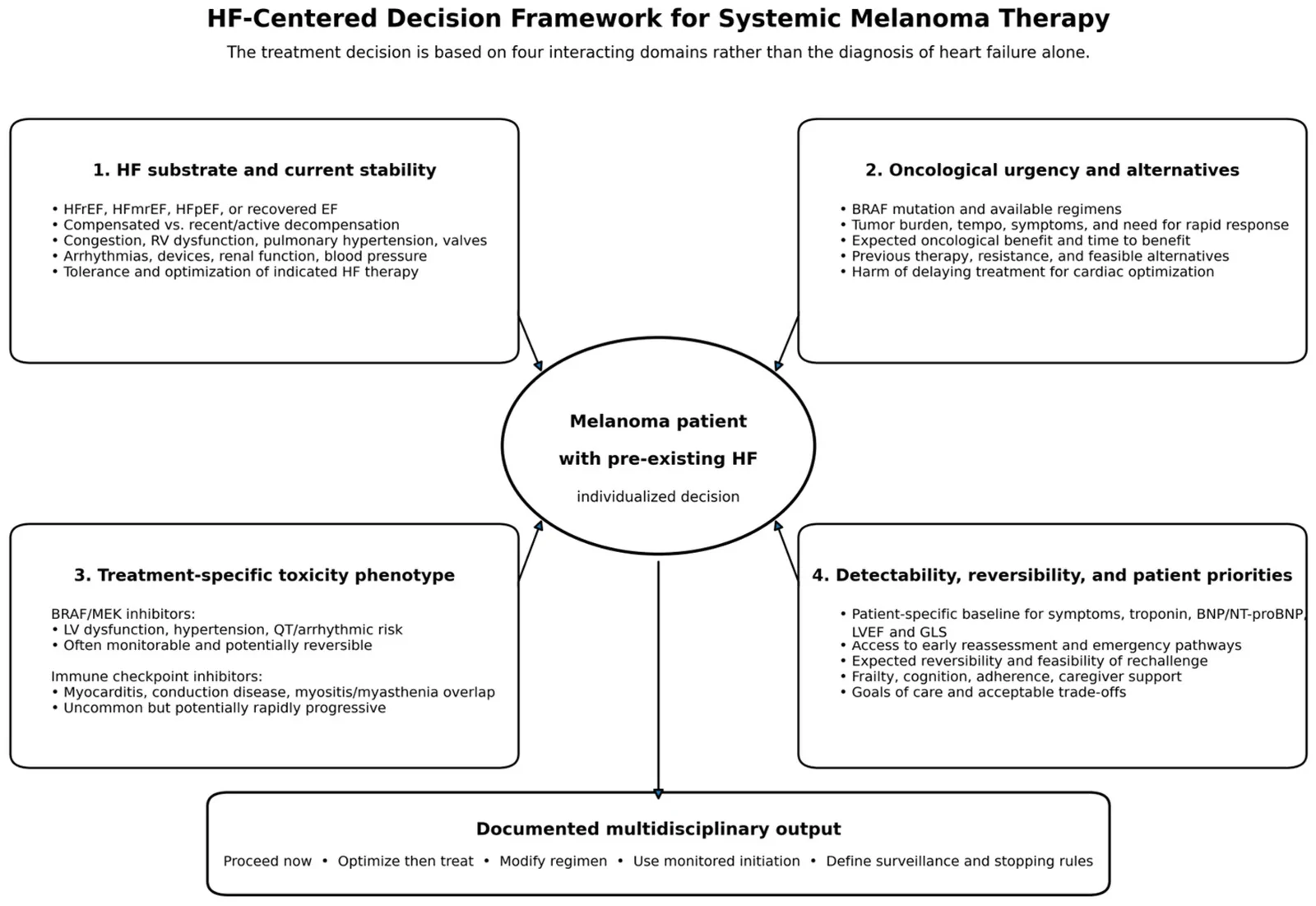
HF-centered decision framework for systemic melanoma therapy. Four interacting domains determine treatment readiness and the intensity of monitoring: (1) HF phenotype and current stability; (2) oncological urgency and alternatives; (3) treatment-specific cardiovascular toxicity phenotype; and (4) detectability, reversibility, frailty, support, and patient priorities. The framework is an author-generated clinical synthesis intended to operationalize existing guidance for patients with pre-existing HF; it is not a validated prediction score. Figure 1 defines the conceptual structure only. It deliberately contains no thresholds, no surveillance intervals and no management steps: the operational sequence from assessment to a documented output is shown in Figure 2, and the therapy-specific management algorithm, including the separate BRAF/MEK inhibitor and checkpoint inhibitor pathways, is shown in Figure 3. The three figures are intended to be read in that order and not to duplicate one another.

To operationalize what is specific to pre-existing HF, we propose a four-domain framework (Figure 1). Domain 1 defines the HF substrate and current stability. Domain 2 defines oncological urgency, expected benefit, time to response, and feasible alternatives. Domain 3 matches the planned regimen to its dominant cardiovascular toxicity phenotype: ventricular dysfunction, hypertension, and QT/arrhythmic risk for BRAF/MEK inhibitors versus myocarditis, conduction disease, and neuromuscular overlap for ICIs. Domain 4 evaluates detectability, reversibility, access to rapid reassessment, frailty, adherence, caregiver support, and patient priorities. The documented output is not simply “treat” or “do not treat,” but one of several calibrated strategies: proceed, optimize then treat, modify the regimen, initiate under enhanced monitoring, or define explicit interruption and rechallenge criteria. This framework complements rather than replaces ESC and IC-OS guidance [3,22].

The four domains are not interchangeable and are not intended to be summed. The grading is deliberately ordinal rather than numerical, because no dataset exists from which quantitative weights could be estimated in this population. Within the framework the domains carry different functions. Domain 1 acts as a gate that determines when treatment may begin. Domain 2 acts as the counterweight that determines how much residual instability is acceptable, and it is the only domain that can override the default to delay. Domain 3 determines what is given and what is measured, but does not by itself contraindicate systemic therapy. Domain 4 acts as a feasibility filter that determines whether the plan generated by Domains 1 to 3 can actually be delivered for this patient. Each domain is graded green, amber, or red against explicit descriptors, and each domain produces its own decision output rather than contributing points to a score (Table 2).

The domain-specific outputs are distinct and should be documented separately. Domain 1 produces a timing decision—treat now, optimize first, or treat only in a monitored setting—together with the baseline dataset that must be recorded before exposure. Domain 2 produces an acceptable delay window expressed in days or weeks, an explicit statement of whether a locoregional, alternative systemic, or best-supportive-care option exists, and a statement of the harm expected from delay. Domain 3 produces the regimen and sequence, the content and timing of surveillance, and the management category assigned to every concomitant cardiovascular medicine. Domain 4 produces the deliverability judgement, the location of care, the patient-specific interruption, resumption and rechallenge criteria, and the goals-of-care record. In older patients it should also produce an explicit record of what the patient is actually trying to achieve: the relative weight given to overall survival as against quality of life, the importance attached to avoiding hospitalization, the willingness to attend frequent monitoring visits, any preference between an oral and an infusion regimen, and the functional threshold below which the patient would not wish treatment to continue. These are recordable items rather than impressions, and they are set out in Section 4.10.

**Figure 2:**
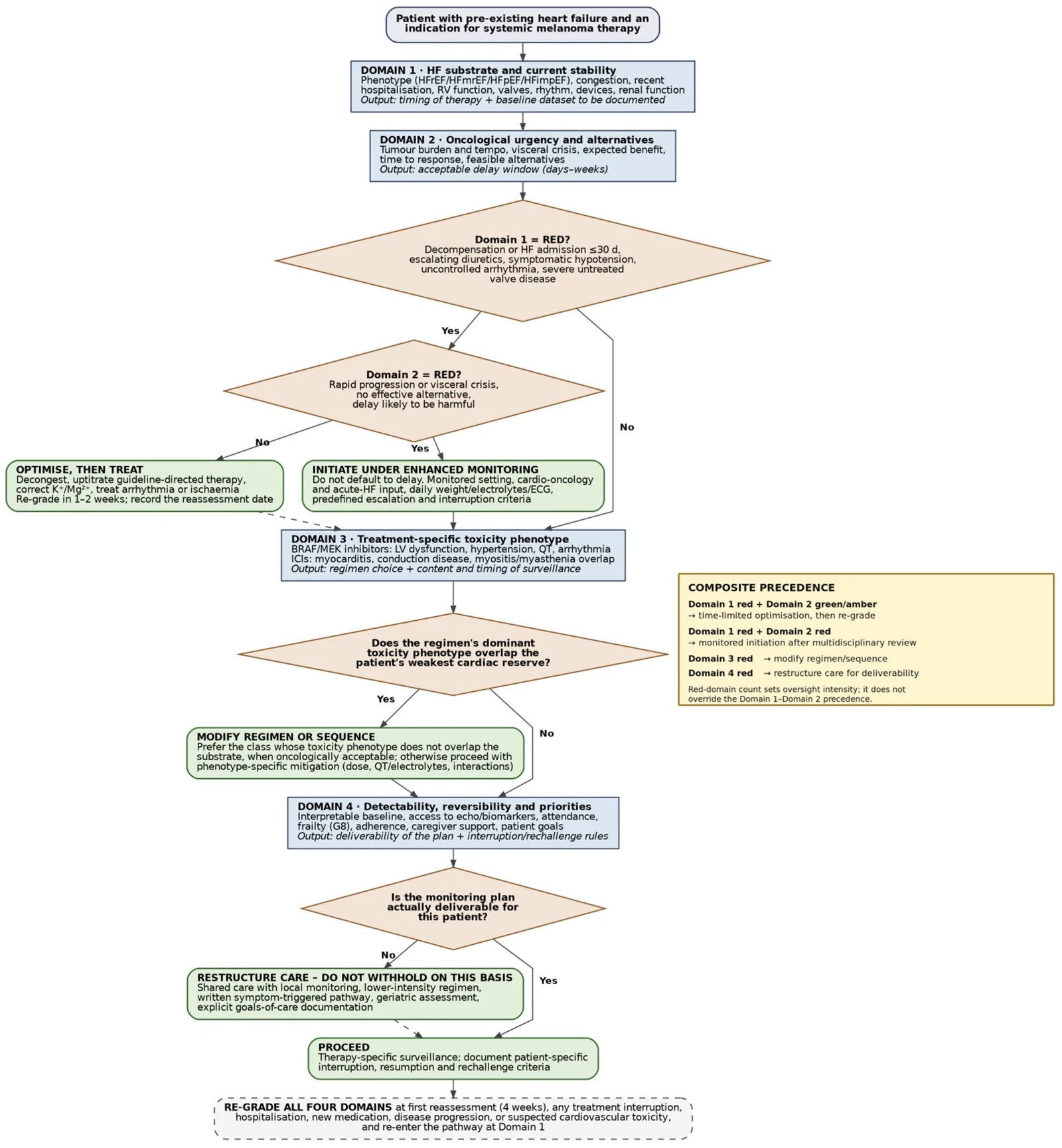
Operational pathway from four-domain assessment to a documented decision output. Blue rectangles denote assessment steps, tan diamonds denote decision nodes, green rounded boxes denote management outputs/actions, and the yellow box summarizes the composite precedence rules. Colors are used to distinguish functional components of the pathway and do not represent the green/amber/red domain grading described in Table 2. Rectangles with a solid outline denote assessment steps, diamonds denote decision nodes, and rounded boxes denote the five possible outputs: proceed; optimize then treat; modify the regimen or sequence; initiate under enhanced monitoring; and restructure care rather than withhold it. The dashed arrow from “Restructure care” to “Proceed” indicates that, once monitoring and care delivery have been made feasible, treatment may proceed rather than being withheld solely because of a Domain 4 deliverability limitation. The composite rule box specifies how simultaneously abnormal domains are resolved, and the dashed box specifies the events that mandate re-grading. The pathway is an author-generated consensus construct that operationalizes existing guidance for patients with pre-existing HF; the thresholds and precedence rules have not been prospectively validated and do not constitute a risk score. ECG, electrocardiography; GDMT, guideline-directed medical therapy; HF, heart failure; HFimpEF, heart failure with improved ejection fraction; ICI, immune checkpoint inhibitor; LV, left ventricular; RV, right ventricular.

Because patients frequently meet criteria in more than one domain simultaneously, the framework specifies how such combinations are resolved rather than leaving the interaction implicit (Figure 2, Table 3). Four precedence rules apply. First, a red Domain 1 with a non-red Domain 2 defaults to optimize-then-treat, with a documented reassessment date rather than an open-ended delay. Second, a red Domain 1 combined with a red Domain 2 does not default to delay; it converts to initiation under enhanced monitoring, because in this specific combination the risk of withholding effective therapy may exceed the cardiovascular risk of administering it. Third, a red Domain 3—meaning that the dominant toxicity phenotype of the planned regimen coincides with the patient’s weakest cardiac reserve—should trigger a change in regimen or sequence when an oncologically acceptable alternative exists, and phenotype-specific mitigation when it does not. Fourth, a red Domain 4 downgrades the plan by one step rather than the patient by one step: a strategy that depends on surveillance that cannot be delivered should be replaced by one that can, not converted into a decision to withhold effective therapy.

The composite pattern is resolved primarily by the interaction between Domains 1 and 2, rather than by the unweighted number of red domains alone. When Domain 1 is red and Domain 2 is green or amber, the default output is time-limited cardiovascular optimization followed by re-grading, irrespective of additional red findings in Domains 3 or 4. When both Domains 1 and 2 are red, treatment should not be delayed solely for cardiovascular optimization; initiation in a monitored setting after multidisciplinary review is preferred. A red Domain 3 modifies the regimen, sequence and surveillance plan, whereas a red Domain 4 requires restructuring of care so that the selected monitoring strategy is deliverable. The number of red domains therefore determines the intensity of multidisciplinary oversight, but does not override the precedence of the Domain 1–Domain 2 interaction. Whenever Domain 2 is red, the record should state explicitly why delay is considered unsafe and what the escalation plan will be, because this is the judgement most likely to be scrutinized retrospectively.

Grading is dynamic rather than a single baseline exercise. All four domains should be re-graded at the first reassessment, at any treatment interruption, after any hospitalization, when a new medicine is introduced, at disease progression, and whenever cardiovascular toxicity is suspected. We emphasize that the descriptors, the ordinal grading, and the precedence rules are author-derived consensus proposals intended to make the decision structure explicit, teachable and auditable. They are neither weighted from outcome data nor prospectively validated, and they should not be used or cited as a risk score. This deserves to be stated in its strongest form, because a systematic presentation can create an impression of systematic derivation that the evidence does not support. The four domains were not extracted from a dataset; they were chosen because they correspond to the four questions that determine what actually happens to the patient. The ordinal descriptors were not fitted to observed outcomes; they are clinical conventions selected to be recognizable at the bedside and reproducible between clinicians. The precedence rules were not tested against an alternative rule or against a comparator; they encode a judgement about which harm is less reversible when two domains are abnormal at once. A structure of this kind can be right or wrong about the anatomy of the problem while remaining entirely silent about the magnitude of any risk, and that is exactly its position here. Its value, if it has any, is that it makes a decision reproducible and auditable within a team, not that it makes the decision correct.

**Table 3:** Resolution of simultaneous domain findings: composite patterns, decision output and minimum monitoring. The composite patterns, decision outputs and minimum monitoring requirements are author-proposed (AP): they operationalize the precedence rules and are intended to be applied together with Figure 2, but they are not derived from outcome data and have not been validated.

Domain Pattern	Composite Category	Decision Output	Minimum Monitoring and Documentation
No red domain	Standard risk for this population	Proceed	Therapy-specific surveillance against the documented baseline; echocardiography at 4 weeks for BRAF/MEK inhibitors in patients above low HFA-ICOS risk; symptom-, ECG- and troponin-triggered review for ICIs.
Domain 1 red; Domain 2 green or amber	Deferrable instability	Optimize, then treat	Decongestion and uptitration of guideline-directed therapy; correction of potassium and magnesium; documented reassessment date within 1–2 weeks; re-grade all four domains before starting.
Domain 1 red; Domain 2 red	Competing risk; delay unsafe	Initiate under enhanced monitoring	Monitored setting with cardio-oncology and acute-HF input; daily weight, electrolytes and ECG; echocardiography at baseline, 2 and 4 weeks; predefined escalation and interruption criteria; explicit written record of why delay was judged unsafe.
Domain 3 red; Domains 1 and 2 non-red	Phenotype mismatch	Modify regimen or sequence	If an oncologically acceptable alternative exists, change class or sequence and record the cardiac rationale; if not, apply phenotype-specific mitigation (dose, QT and electrolyte management, interaction handling) with earlier reassessment.
Domain 4 red; other domains non-red	Deliverability failure	Restructure care; do not withhold on this basis	Shared care with local monitoring; lower-intensity regimen where oncologically reasonable; written symptom-triggered pathway and direct contact route; geriatric assessment when indicated; explicit goals-of-care documentation.
Domain 1 red and Domain 2 red, with ≥1 additional red domain	Very high composite risk; delay unsafe	Monitored initiation only, after multidisciplinary review	Monitored-setting measures specified above, plus formal multidisciplinary documentation, a resuscitation and device plan, and a recorded goals-of-care discussion. The record must state why oncological delay was judged unsafe.
≥2 red domains with Domain 2 green	Unfavorable balance at this time	Defer and optimize	Time-limited optimization with a defined re-grading date; reconsider locoregional or non-systemic options; document that this is a temporary rather than a permanent exclusion.
Any domain re-graded to red during treatment	Dynamic escalation	Interrupt or intensify monitoring according to severity	Attribute the event explicitly (progression of underlying HF, treatment-associated ventricular dysfunction, or myocarditis); apply IC-OS severity language; define resumption and rechallenge criteria before restarting.

**Figure 3:**
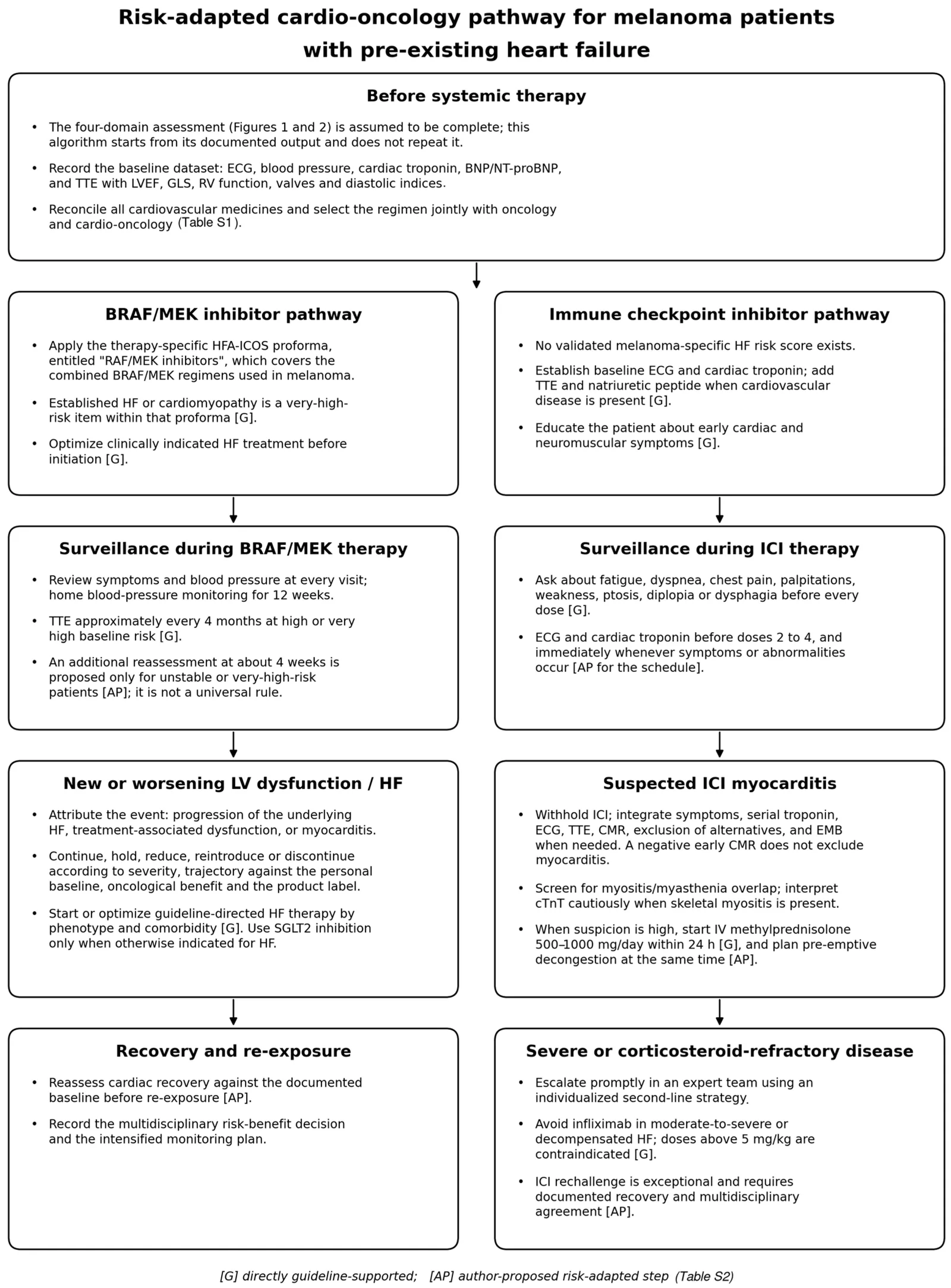
Proposed therapy-specific cardio-oncology pathway for melanoma patients with pre-existing heart failure. Read from top to bottom: the left column follows BRAF/MEK inhibitor management and the right column follows immune checkpoint inhibitor management. Figure 1 provides the conceptual four-domain framework and Figure 2 provides the decision logic; Figure 3 begins only after that assessment is complete. Steps are labeled [G] when directly guideline-supported and [AP] when author-proposed. CMR, cardiac magnetic resonance; CTRCD, cancer therapy-related cardiac dysfunction; EMB, endomyocardial biopsy; GDMT, guideline-directed medical therapy; IVIG, intravenous immunoglobulin; MDT, multidisciplinary team; TTE, transthoracic echocardiography.

#### 3.1.3. HF Phenotype, Treatment Readiness, and Monitoring Consequences

Stable chronic HF is not, by itself, a contraindication to BRAF/MEK inhibition or ICI therapy. In contrast, active congestion, escalating diuretic requirement, recent HF hospitalization, hypotension, progressive renal dysfunction, uncontrolled arrhythmia, or untreated severe valvular disease should prompt stabilization before non-emergent treatment. When melanoma is rapidly progressive and delay is likely to be harmful, therapy may still be initiated in a monitored setting with predefined cardiovascular support and reassessment. Table 4 summarizes how common HF substrates alter baseline interpretation and management; these proposals are consensus-based because comparative melanoma data across HF phenotypes do not exist.

#### 3.1.4. Heart Failure with Preserved Ejection Fraction: Deterioration Without a Change in Ejection Fraction

HFpEF accounts for approximately half of all prevalent HF and its share rises steeply with age, obesity, type 2 diabetes, atrial fibrillation and chronic kidney disease [23]—that is, with exactly the profile of the older patient who now presents with advanced melanoma. It is also the phenotype in which an ejection-fraction-centered surveillance strategy is most likely to fail, because LVEF is normal at baseline, remains normal during deterioration, and therefore cannot function as the change variable. A patient may develop progressive congestion, rising filling pressures, a fall in functional capacity and even an HF hospitalization during systemic melanoma therapy while every recorded LVEF stays above 50%. Surveillance built solely on serial ejection fraction will record such an episode as an absence of cardiotoxicity, and the IC-OS categories, which are anchored on LVEF and on a relative fall in global longitudinal strain, will not classify it as an event [22].

The practical consequence is that in HFpEF the monitored variables, and not merely the monitoring interval, must change (author-proposed). We suggest that the baseline dataset include, in addition to LVEF and global longitudinal strain, an assessment of diastolic function and estimated filling pressures, left atrial volume index, estimated pulmonary artery systolic pressure with tricuspid regurgitation velocity, right ventricular systolic function including the tricuspid annular plane systolic excursion and its ratio to the estimated pulmonary artery systolic pressure where obtainable, valvular severity, rhythm and atrial fibrillation burden, body weight and diuretic requirement, blood pressure, renal function, and a documented natriuretic-peptide value, together with an objective statement of exercise tolerance rather than NYHA class alone. Follow-up then tracks the trajectory of these variables against that baseline. In the absence of validated criteria we propose the following triggers for reassessment, any one of which should prompt clinical review even when the ejection fraction is unchanged: a sustained rise in NT-proBNP above the patient’s own baseline that is not explained by an intercurrent illness; any increase in diuretic requirement; weight gain with orthopnea or peripheral oedema; new atrial fibrillation or a faster ventricular response; a fall in estimated glomerular filtration rate accompanying either congestion or aggressive decongestion; a deterioration in functional class or exercise tolerance; and a new oxygen requirement.

Several features of systemic melanoma therapy interact specifically with this substrate. MEK inhibitor-associated hypertension raises afterload in a ventricle that is already stiff, and it is worth noting that the only prospective cardiovascular cohort in melanoma reported treatment-associated hypertension in 45.9% of participants, a proportion far exceeding that of moderate or severe systolic dysfunction [13]; in HFpEF, hypertension is not a lesser toxicity than a fall in ejection fraction but a more relevant one. Diarrhea, vomiting, pyrexia and reduced oral intake produce hypovolemia and acute kidney injury in a diuretic-dependent patient, whereas the sodium and water retention caused by high-dose corticosteroids given for immune-related toxicity produces the opposite problem; the stiff ventricle tolerates errors in either direction poorly because it operates on the steep portion of the pressure–volume relationship. Atrial fibrillation with a rapid ventricular response shortens diastolic filling time and can precipitate decompensation with no myocardial injury at all, which matters because atrial arrhythmia is among the reported pharmacovigilance signals for BRAF/MEK inhibitors [12] and because new arrhythmia during checkpoint blockade must simultaneously raise the possibility of myocarditis. Finally, obesity, insulin resistance, systemic inflammation and impaired sodium handling are not incidental comorbidities in HFpEF but constituents of its mechanism, and they also lower natriuretic-peptide concentrations for any given filling pressure; so, a “normal” value is less reassuring in an obese patient than in a lean one.

Within the four-domain framework, the implication is that a preserved ejection fraction must never be entered as a green Domain 1. A congestion-prone patient with HFpEF, atrial fibrillation and chronic kidney disease is graded amber or red on the same stability criteria as a patient with HFrEF, and the decision output is correspondingly the same: an interpretable multi-parameter baseline before exposure, and a surveillance plan whose triggers are congestion, natriuretic-peptide trajectory, renal function, rhythm and functional capacity rather than ejection fraction alone (author-proposed). Case 4 in Section 3.7 illustrates this grading in a patient in whom the preserved ejection fraction is the least informative of the available measurements.

### 3.2. BRAF/MEK Inhibitors: Evidence Relevant to Pre-Existing HF

#### 3.2.1. Mechanistic Context and HF Relevance

BRAF and MEK inhibitors suppress the RAS–RAF–MEK–ERK pathway, which supports cardiomyocyte adaptation to mechanical and ischemic stress [36]. In a patient with established HF, reduced reserve provides a biologically plausible reason for greater clinical consequences from additional ventricular dysfunction, hypertension, or arrhythmia; however, this susceptibility has not been quantified prospectively. Mechanistic plausibility should therefore inform vigilance, not be presented as proof of excess HF-specific risk.

#### 3.2.2. Observational Incidence and Limits of Applicability

Reported BRAF/MEK inhibitor-associated cardiac dysfunction varies with regimen, event definition, baseline selection, and surveillance intensity. Quantitative estimates are derived predominantly from recent longitudinal and real-world cohorts rather than randomized trials with adjudicated cardiovascular endpoints [9,10,11,12,13]. Because established HF was uncommon, excluded, or not analyzed as a dedicated subgroup, these values describe treated melanoma populations and are not HF-specific incidence estimates.

Table 5 summarizes the design, sample size, regimen composition, surveillance schedule, follow-up duration and event definition of five representative sources, together with the cardiac outcomes reported by each. Across the three retrospective real-world cohorts, reported cardiac dysfunction ranged from 18% to 27%, while events classified as major or moderate occurred in approximately 6% to 14% [9,10,11]. In the 170-patient cohort, 79% of cardiotoxicity cases were reported as reversible after dose reduction, treatment interruption, HF therapy, or continued treatment with monitoring [9]. The only prospective cohort, which applied protocolized echocardiography with strain, home blood-pressure monitoring and biomarkers at baseline and at 4, 12 and 24 weeks, reported CTRCD in 45.9% of participants; however, 85.7% of these cases were mild, moderate or severe CTRCD occurred in 6.6% of the cohort overall, every moderate or severe case was already apparent at 4 weeks, and no participant developed symptomatic HF [13]. Pharmacovigilance analysis broadens the recognized phenotype to hypertension, arrhythmia, QT abnormalities and thromboembolic signals, but provides no denominator and cannot estimate incidence [12].

The apparent spread between 18% and 46% is therefore largely an artefact of case definition and ascertainment rather than a difference in underlying biology. Cohorts that counted only ejection-fraction thresholds and imaged every three months report lower figures than a prospective cohort that also counted a relative fall in global longitudinal strain and imaged at four weeks. Displaying these values side by side as a single bar chart would imply a comparability that does not exist, which is why they are tabulated together with the characteristics that generate the difference. Equally important, none of these studies was designed to describe patients with established HF: pre-existing left ventricular systolic dysfunction was present in a single participant (1.6%) in the prospective cohort, which also required performance status 0–1 and a predicted survival of at least six months, and none of the retrospective cohorts reported a dedicated HF stratum. These data therefore support surveillance and potential reversibility, but they do not establish the comparative safety of BRAF/MEK therapy across HFrEF, HFpEF, recent decompensation, or advanced HF.

ICI-associated myocarditis should not be placed alongside these figures as an equivalent endpoint. Typical clinical estimates are approximately 0.3–1.1%, whereas higher values, including estimates approaching 4%, have been reported in selected or intensively screened populations [15,18,21]. The denominators, definitions and consequences differ so substantially that any direct numerical comparison between BRAF/MEK-associated ventricular dysfunction and ICI-associated myocarditis is misleading.

#### 3.2.3. Risk Markers in the Setting of Pre-Existing HF

In a prospective real-world cohort, hypertension and/or CTRCD was detected frequently during BRAF/MEK inhibitor therapy, and moderate or severe abnormalities appeared early in patients with at least medium baseline risk; elevated baseline NT-proBNP was associated with incident CTRCD [13]. These findings support early risk-adapted surveillance but do not establish a universal four-week imaging requirement or a validated prediction model for patients with established HF.

Potential risk markers include known HF or cardiomyopathy, reduced baseline LVEF, hypertension, ischemic heart disease, older age, and abnormal cardiac biomarkers. However, most associations arise from small or unselected cohorts, and the incremental risk attributable to established HF has not been quantified. Baseline LVEF and GLS alone may not reliably identify all patients who later develop dysfunction; clinical status, chamber dimensions, biomarkers, blood pressure, and treatment exposure should be interpreted together.

#### 3.2.4. Clinical Presentation, Reversibility, and Attribution

The clinical presentation of BRAF/MEK inhibitor-associated CTRCD is heterogeneous and often asymptomatic, posing diagnostic challenges. A case report described a 50-year-old woman who developed CTRCD attributable to combined BRAF and MEK inhibition for malignant melanoma, presenting atypically with isolated severe fatigue and without classical heart failure symptoms; echocardiographic and cardiac magnetic resonance (CMR) imaging revealed a pattern consistent with apical hypertrophic cardiomyopathy—a morphological finding rare in Central Europe—which completely resolved on follow-up imaging after discontinuation of targeted therapy and initiation of ACE inhibitor, beta-blocker, and SGLT2 inhibitor therapy. This case emphasizes that CTRCD may mimic other cardiomyopathies and that advanced imaging modalities are indispensable in the diagnostic workup [8].

### 3.3. Immune Checkpoint Inhibitors in Patients with Pre-Existing HF

#### 3.3.1. Mechanistic Context

ICI therapy restores antitumor T-cell activity but can disrupt cardiac immune tolerance. Proposed mechanisms include expansion of autoreactive T cells, cross-reactivity between tumor and myocardial antigens, and cytokine-amplified inflammation [14,15]. For patients with HF, the key clinical distinction is not a proven higher incidence of myocarditis, which remains unknown, but a lower tolerance of conduction disease, arrhythmia, inflammatory myocardial injury, fluid shifts, and respiratory-muscle weakness.

#### 3.3.2. Incidence, Severity, and HF-Specific Uncertainty

ICI-associated myocarditis is uncommon. Most clinical series and registries report an incidence of approximately 0.3–1.1%, although the estimate varies with case definition, treatment regimen, population, and surveillance intensity [15,18,21]. These estimates arise mainly from multicenter case series, registries, observational cohorts, and pharmacovigilance analyses rather than randomized melanoma trials with systematic myocarditis adjudication. Values approaching 4% have been described in selected or intensively screened cohorts and should not be presented as the expected incidence in routine unselected practice. Combination ICI therapy and intensive biomarker surveillance may increase case detection, including milder or subclinical presentations.

ICI-associated myocarditis usually occurs early, often during the first two to three months, and is more frequent after combination checkpoint blockade [16,17,18]. Clinical severity is heterogeneous and may include myocyte injury, conduction disease, ventricular arrhythmia, HF, or cardiogenic shock. Reported case-fatality estimates vary according to ascertainment and case severity; historical series have described mortality of approximately 27–50%, but these values should not be interpreted as uniform across contemporary cohorts [17,18].

A landmark multicenter case series drawing on six clinical centers documented a broad spectrum of cardiovascular toxicities following ipilimumab and/or nivolumab/pembrolizumab administration, including heart failure, cardiomyopathy, complete heart block, myocardial fibrosis, and myocarditis [18]. A systematic analysis of published case reports identified male sex, lung cancer, melanoma, and treatment with anti-PD-1 monotherapy or anti-PD-1 combined with anti-CTLA-4 as factors potentially increasing the risk of ICI-associated myocarditis [19].

#### 3.3.3. Diagnosis When HF Abnormalities Pre-Date Therapy

ICI myocarditis may present with dyspnea, chest pain, fatigue, palpitations, presyncope, HF, ventricular arrhythmia, or atrioventricular block. Diagnosis requires integration of symptoms, serial cardiac troponin changes, ECG, echocardiography, CMR, and exclusion of acute coronary syndrome, infection, pulmonary embolism, tumor progression, and other causes of myocardial injury. Both cardiac troponin I (cTnI) and troponin T (cTnT) are informative; however, cTnT may also rise in immune-mediated skeletal myositis; so, an apparently discordant cTnT elevation should prompt neuromuscular assessment rather than automatic attribution to myocarditis. cTnI may offer greater cardiac specificity in this context, but neither assay should be interpreted in isolation [22,41,42,43,44].

CMR is an important diagnostic modality, and the updated Lake Louise framework can provide major diagnostic evidence [3,22]. Nevertheless, late gadolinium enhancement and edema may be absent, especially early in the course; therefore, a negative or nondiagnostic CMR does not exclude ICI myocarditis [41]. Endomyocardial biopsy remains the histological reference standard and should be considered when the diagnosis remains uncertain, when noninvasive tests conflict, or in severe or refractory presentations. Because myocarditis may overlap with myositis and myasthenia gravis, patients should be assessed for proximal or respiratory muscle weakness, myalgia, ptosis, diplopia, dysphagia, dysarthria, CK elevation, and ventilatory compromise; neurologic input, respiratory measurements, and antibody/electrophysiological testing may be required [43,45].

#### 3.3.4. Direct Clinical Evidence in Pre-Existing HF

Direct evidence is currently limited to case-level experience. A 2024 report described a 42-year-old man with BRAF V600E-mutated melanoma and NYHA class III HF who received nivolumab/ipilimumab followed by encorafenib/binimetinib after multidisciplinary assessment; tumor response was accompanied by improvement in HF without major treatment-induced cardiotoxicity [7]. The report shows that systemic therapy can be feasible and that malignant effusions or tumor-related hemodynamic effects may mimic or aggravate HF, but it cannot define incidence, comparative safety, or a preferred regimen. No prospective cohort has reported outcomes stratified by established HF phenotype and stability.

### 3.4. Drug–Drug Interactions in the HF Medication Context

#### 3.4.1. Pharmacokinetic Interactions with BRAF/MEK Inhibitors

Drug–drug interaction assessment must be regimen-specific. BRAF and MEK inhibitors differ in their dependence on CYP3A4, CYP2C8, transporters, and their capacity to induce or inhibit metabolic pathways. Therefore, a cardiovascular medicine should not be labeled “contraindicated” solely because it is a CYP substrate or a weak/moderate inhibitor. Interaction direction, magnitude, dose, QT liability, therapeutic index, and the individual product label must be considered [30,31,32,33,34,35,38,39,40].

The prior statement that amlodipine, nifedipine, amiodarone, dronedarone, atorvastatin, lovastatin, simvastatin, and proton-pump inhibitors are uniformly contraindicated is not supported by official prescribing information. For example, dabrafenib exposure increases with strong CYP3A4 or CYP2C8 inhibitors, whereas dabrafenib also induces CYP3A4 and CYP2C9 and can reduce exposure to substrates such as midazolam and warfarin; acid-reducing therapy does not produce a clinically relevant change in dabrafenib exposure [30]. Encorafenib requires avoidance or dose modification with strong or moderate CYP3A4 inhibitors and avoidance of QT-prolonging medicines, while binimetinib has comparatively limited direct pharmacokinetic interaction liability [32,33].

Amiodarone and dronedarone merit particular caution when the melanoma regimen itself prolongs QT or depends on CYP3A4, but the action may be avoidance, substitution, dose adjustment, or enhanced ECG/electrolyte monitoring rather than a universal contraindication. Amlodipine and nifedipine may have reduced exposure with strong CYP3A induction, potentially requiring blood-pressure monitoring and dose adjustment. Statin selection should consider CYP3A and transporter pathways; pravastatin or another less CYP3A-dependent option may be preferable in some regimens, whereas rosuvastatin exposure may increase transiently with dabrafenib. Warfarin requires close INR monitoring because enzyme induction can reduce anticoagulant exposure [30,31,32,33,34,35,40], as shown in Table S1.

A structured medication review should be repeated at treatment initiation, dose modification, hospital discharge, and any new prescription. Official product information and a recognized oncology interaction database should be checked for the exact drug pair, and the management category should be documented as contraindicated/avoid, dose-adjust, monitor closely, or no clinically important interaction expected. The Drug-PIN and traffic-light approaches may help identify complex polypharmacy, but they do not replace agent-specific verification [38,39,40].

#### 3.4.2. Pharmacodynamic and HF-Treatment Considerations

Pharmacodynamic effects require separate consideration from metabolism. Diuretics may worsen hypokalemia or hypomagnesemia and amplify QT risk. Bradycardia from beta-blockers or antiarrhythmics may compound conduction or QT vulnerability, but beta-blockers should not be withheld automatically when indicated for HF. RAAS inhibitors, mineralocorticoid-receptor antagonists, and SGLT2 inhibitors should be managed according to blood pressure, renal function, potassium, volume status, and the patient’s HF phenotype. The appropriate response is individualized monitoring and dose adjustment rather than categorical avoidance.

#### 3.4.3. Special Considerations for Heart Failure Medication During Systemic Melanoma Therapy

Interaction screening answers only half of the question in a patient who is already established on HF therapy. The other half is how that therapy should itself be managed during melanoma treatment. The default position should be continuation of guideline-directed medical therapy, because withdrawing disease-modifying HF treatment during a period of additional cardiovascular stress is itself a hazard, and none of the regimens considered here requires routine discontinuation of any HF drug class [23,24,25]. The adjustments that are genuinely required are mostly pharmacodynamic and situational rather than absolute, and they are summarized in Table 6.

Renin–angiotensin system inhibitors and angiotensin receptor–neprilysin inhibitors have no clinically important cytochrome-mediated interaction with BRAF/MEK inhibitors or ICIs and should be continued. The practical risks are hypotension and acute kidney injury during the diarrhea, vomiting, pyrexia and reduced oral intake that accompany both treatment classes; so, temporary dose reduction with early re-uptitration is preferable to permanent withdrawal. One point is specific to surveillance in this population: because sacubitril inhibits neprilysin and therefore raises B-type natriuretic peptide while N-terminal pro-B-type natriuretic peptide remains interpretable, only NT-proBNP should be used to track treatment-associated change in a patient receiving an ARNI. Mineralocorticoid-receptor antagonists should likewise be continued, with potassium and renal function checked at each cycle; eplerenone is a sensitive CYP3A4 substrate; so, where a strong CYP3A4 inhibitor cannot be avoided, substitution with spironolactone or dose reduction is more appropriate than abandoning mineralocorticoid blockade [30,31,32,33,34,35,40].

Beta-blockers should not be withheld or pre-emptively reduced in HFrEF. Their presence does, however, change the interpretation of new bradycardia or atrioventricular block during checkpoint blockade: new conduction disease in a patient receiving an ICI should be treated as possible myocarditis and investigated as such, rather than attributed to the beta-blocker and managed by dose reduction alone. Bisoprolol undergoes partial CYP3A4 metabolism, and carvedilol, metoprolol and nebivolol are CYP2D6 substrates; so, enzyme induction by dabrafenib or encorafenib may reduce exposure; the clinical response should therefore be judged by heart rate and blood pressure rather than by the prescribed dose. Digoxin is a P-glycoprotein substrate with a narrow therapeutic index and its exposure may increase with vemurafenib or encorafenib; so, concentrations should be checked and hypokalemia avoided. Ivabradine is a sensitive CYP3A4 substrate with additional bradycardic and QT liability and is best avoided with encorafenib-containing regimens. In device-bearing patients, interrogation should be performed at baseline, after any suspected myocarditis and before cardiac magnetic resonance, and the magnetic-resonance-conditional status of the device should be confirmed in advance so that imaging is not delayed at the moment it is most needed [30,31,32,33,34,35,40].

Sodium–glucose cotransporter 2 inhibitors have no significant cytochrome interaction and should be continued when indicated, but they warrant explicit sick-day rules: temporary withholding during significant diarrhea, vomiting, fasting, sepsis or the perioperative period is appropriate, because the combination of reduced intake, cancer-associated catabolism and volume depletion is the setting in which euglycemic ketoacidosis occurs. Loop diuretics require the opposite discipline: the dose should follow daily weight and congestion rather than remain fixed, and potassium and magnesium should be measured and repleted before and during any QT-prolonging regimen, since diuretic-induced electrolyte depletion, antiemetics such as ondansetron, and encorafenib or vemurafenib are additive on the QT interval. Anticoagulation deserves particular attention because BRAF/MEK regimens carry a thromboembolic signal [12]: warfarin exposure falls with enzyme induction and requires close INR monitoring, apixaban and rivaroxaban are CYP3A4 and P-glycoprotein substrates whose concentrations may fall with dabrafenib, encorafenib or vemurafenib, and low-molecular-weight heparin is a reasonable choice when the interaction is unpredictable and the thrombotic risk is high.

Finally, the treatment of immune-related toxicity has HF consequences of its own. High-dose methylprednisolone causes sodium and water retention, hypertension, hyperglycemia and hypokalemia, and in a patient with HFrEF it may precipitate congestion at precisely the moment when myocardial reserve is lowest. Pre-emptive diuretic uptitration, daily weights, and daily potassium and glucose monitoring should therefore be planned at the same time as corticosteroids are started rather than after decompensation occurs [20]. Infliximab should generally be avoided in this population, and doses above 5 mg/kg are contraindicated in moderate or severe HF [46,47].

### 3.5. HF-Specific Risk Stratification and Monitoring

#### 3.5.1. Therapy-Specific HFA-ICOS Assessment

The 2022 ESC Cardio-Oncology Guidelines recommend baseline cardiovascular assessment and therapy-specific risk stratification before potentially cardiotoxic treatment [3]. The HFA-ICOS proformas were developed for specific treatment classes rather than as a single universal score [26]. They organize clinical history, cardiovascular disease, risk factors, biomarkers, and imaging findings, but prospective validation in melanoma remains limited and observed events can still occur in patients categorized as low or medium risk [10].

For combined BRAF/MEK inhibitor therapy, established HF or cardiomyopathy is a very-high-risk factor within the corresponding HFA-ICOS baseline risk proforma, which is entitled “RAF/MEK inhibitors” in the original position statement and refers to the same approved combinations [26]. This classification should not be generalized automatically to every ICI regimen, for which no equivalently validated melanoma-specific HF risk score exists. In practice, any patient with active or previously decompensated HF warrants cardio-oncology review, but the intensity of surveillance should be linked to the planned therapy, HF stability, baseline LVEF/GLS and biomarkers, prior cardiovascular events, and competing oncological urgency.

#### 3.5.2. Interpreting Surveillance Against an Abnormal Baseline

Surveillance should be therapy-specific and adaptive. For BRAF/MEK inhibitors, clinical review, blood-pressure control, symptom assessment, ECG when indicated, and serial ventricular-function assessment are reasonable. For ICIs, early recognition of symptoms, ECG abnormalities, and troponin changes is central because myocarditis usually occurs early. Biomarkers are most interpretable when a reliable baseline is available; persistently abnormal values in chronic HF require comparison with the patient’s baseline and clinical trajectory rather than a binary threshold.

Patients with pre-existing HF should undergo baseline echocardiography including LVEF, GLS when feasible, right ventricular function, valves, and diastolic parameters, together with ECG, blood pressure, troponin, and BNP/NT-proBNP [3,26,48]. For high- or very-high-risk BRAF/MEK therapy, ESC-consistent surveillance may include echocardiography approximately every four months; the exact interval should be adapted to clinical status and the product label [3,10,36]. An earlier reassessment at approximately four weeks may be reasonable in unstable or very-high-risk patients and is supported by prospective observational protocols [13], but it is an author-proposed risk-adapted strategy rather than a universal guideline mandate. New symptoms, biomarker dynamics, blood-pressure deterioration, or treatment changes should trigger earlier evaluation.

Because the two treatment classes differ in the organ manifestation, the time course and the reversibility of their cardiovascular toxicity, a single generic surveillance schedule is not clinically useful. The corresponding assessments are therefore set out separately in Table 7, which distinguishes what should be obtained before the first exposure from what should be repeated during treatment with a BRAF/MEK inhibitor and during checkpoint blockade, and which identifies for each item whether it is directly guideline-supported or author-proposed. Two asymmetries are worth stating explicitly. During BRAF/MEK inhibitor therapy the principal instruments are blood pressure and serial ventricular function, and troponin is a problem-solving rather than a scheduled test; during checkpoint blockade the principal instruments are the symptom review, the ECG and serial troponin in the first twelve weeks, and echocardiography is a problem-solving test, because myocarditis frequently occurs with a preserved ejection fraction.

### 3.6. Management When HF Pre-Exists

#### 3.6.1. New or Worsening Ventricular Dysfunction During BRAF/MEK Therapy

Management of BRAF/MEK inhibitor-associated CTRCD should use IC-OS severity definitions as a common language but should not be reduced to rigid interruption thresholds [22]. The decision to continue, temporarily withhold, reduce, reintroduce, or permanently discontinue treatment should integrate HF symptoms and congestion, absolute LVEF and GLS change, trajectory on repeat imaging, arrhythmia or ischemia, alternative causes, oncological benefit, expected reversibility, and the dose-modification instructions for the specific BRAF/MEK pair [3,30,31,32,33,34,35]. Mild asymptomatic dysfunction may permit continuation with intensified monitoring and HF therapy. Moderate dysfunction often prompts temporary interruption or dose reduction, but continued treatment can be considered in selected stable patients after multidisciplinary review. Severe, symptomatic, progressive, or non-recovering dysfunction generally favors interruption and may require discontinuation; permanent discontinuation is not automatic solely from a severity label when the product information allows for recovery-guided reintroduction. The evidence basis of this paragraph should be read as follows: the IC-OS severity vocabulary and the dose-modification instructions of each product label are guideline-supported [22,30,31,32,33,34,35], whereas the proposal to reference every threshold to the patient’s own baseline and trajectory rather than to a population cut-point, and the resumption criteria described here, are author-proposed and have not been validated in any prospective study.

HF treatment should follow contemporary guideline-directed therapy according to LVEF phenotype, symptoms, blood pressure, renal function, potassium, and comorbidities [23,24,25]. ACE inhibitors/ARBs or ARNI, evidence-based beta-blockers, mineralocorticoid-receptor antagonists, diuretics, and SGLT2 inhibitors should be used when clinically indicated; none has been proven in a prospective trial specifically to prevent or reverse BRAF/MEK inhibitor cardiotoxicity. A published case demonstrated recovery after treatment withdrawal and multidrug HF therapy that included an SGLT2 inhibitor [8]; however, this patient did not have pre-existing HF, and causality cannot be assigned to any single component. SGLT2 inhibition is therefore promising and appropriate when independently indicated for HF, not an established toxicity-specific cardioprotective intervention.

#### 3.6.2. Suspected ICI Myocarditis and HF Decompensation

ICI treatment should be withheld immediately when myocarditis is clinically suspected, and the patient should undergo urgent ECG, serial troponin, echocardiography, rhythm monitoring, and further multimodal evaluation according to severity [3,22]. When suspicion is high, intravenous methylprednisolone 500–1000 mg/day should be initiated as early as possible—ideally within 24 h—without waiting for CMR or biopsy confirmation in an unstable patient [3,20,49]. Early high-dose treatment has been associated observationally with fewer major adverse cardiovascular events, although randomized evidence is unavailable [49]. Subsequent ICI discontinuation or rare rechallenge should be decided after defining diagnostic certainty, myocarditis severity, cardiac recovery, cancer status, and alternatives.

Failure of troponin, rhythm, conduction, ventricular function, or clinical status to improve promptly despite high-dose corticosteroids should trigger expert reassessment and consideration of additional immunosuppression. There is no validated universal definition based solely on “no recovery within three days,” and second-line agents are not interchangeable. Selection should reflect hemodynamic severity, conduction/arrhythmic complications, concomitant myositis or myasthenia, infection risk, organ function, and local expertise [45,50,51]. Table 8 summarizes the evidence and HF-specific cautions. Infliximab has only sparse off-label evidence in ICI myocarditis and carries a major HF safety concern; it should generally be avoided in moderate-to-severe or decompensated HF, and doses above 5 mg/kg are contraindicated in moderate or severe HF according to prescribing information [46,47].

ICI rechallenge after myocarditis is exceptional. It should be considered only after multidisciplinary review in a patient with strong oncological need, high diagnostic confidence regarding the prior event, documented cardiovascular recovery, and a feasible intensive monitoring plan. Severe or complicated myocarditis generally argues against rechallenge (Figure 3). The decision should be individualized rather than framed as routinely permissible or uniformly prohibited [3]. Here too the components differ in status: immediate withholding of the checkpoint inhibitor, urgent multimodal assessment and early high-dose methylprednisolone are guideline-supported [3,20,22,49], whereas the specific rechallenge preconditions proposed above—documented diagnostic certainty about the index event, demonstrated cardiovascular recovery against the personal baseline, and a deliverable intensive monitoring plan—are author-proposed and are offered as a documentation standard rather than as evidence of safety.

### 3.7. Illustrative Application of the Four-Domain Framework

Because the direct evidence in this population is case-level, the framework is most usefully examined against concrete clinical situations. The four vignettes below are illustrative composites constructed by the authors from published case-level reports [7,8], the cohort observations summarized in Table 5, guideline recommendations and prescribing information. They do not describe identifiable individual patients and were not derived from clinical records. Each vignette states the grade assigned to every domain, the resulting decision output, and the reassessment that followed; the four cases are compared in Table 9.

#### 3.7.1. Case 1: Stable HFrEF and BRAF V600E-Mutated Melanoma Without Visceral Crisis

A 68-year-old man with ischemic HFrEF (LVEF 38%, NYHA class II) has been stable for 18 months on sacubitril/valsartan, bisoprolol, spironolactone and dapagliflozin, with an NT-proBNP of approximately 900 pg/mL that has not changed over a year. He presents with BRAF V600E-mutated metastatic melanoma, performance status 1, moderate tumor burden and no visceral crisis. Domain 1 is amber: the substrate is abnormal and reserve is reduced, but nothing is unstable; so, no delay is required and the requirement is documentation of an interpretable baseline (LVEF, GLS, RV function, valves, ECG, troponin, NT-proBNP, renal function, electrolytes). Domain 2 is amber: therapy is clearly indicated, but a one- to two-week window for baseline assessment is safe and an alternative class exists. Domain 3 is red for combined BRAF/MEK inhibition, because ventricular dysfunction, hypertension and QT prolongation are precisely the toxicities this substrate tolerates least and established cardiomyopathy is a very-high-risk item in the corresponding HFA-ICOS proforma [26]; it is amber for checkpoint blockade, since there is no prior myocarditis and no conduction disease. Domain 4 is green. With a single red domain that is regimen-specific rather than patient-specific, the output is not “do not treat” but “modify the sequence”: checkpoint blockade first—a choice that is also supported at population level [37]—with baseline and early ECG and troponin, a written symptom-triggered pathway, and BRAF/MEK inhibition retained as a second-line option with a mandated four-week echocardiogram if it is used.

#### 3.7.2. Case 2: Recent Decompensation with Rapidly Progressive Melanoma

A 59-year-old woman is discharged twelve days after a HF admission. She has HFmrEF (LVEF 42%), permanent atrial fibrillation on amiodarone and apixaban, moderate mitral regurgitation, and an NT-proBNP that has fallen from 4800 to 2600 pg/mL on an oral diuretic regimen that is not yet stable. She has BRAF V600E-mutated melanoma with symptomatic liver metastases, a rising lactate dehydrogenase and performance status 2. Domain 1 is red (decompensation within 30 days, unstable diuretic requirement). Domain 2 is also red: progression is rapid, organ function is threatened, and an immunotherapy-first sequence, although supported at population level, has a slower expected time to response than this patient can tolerate. Domain 3 is red for the class that is oncologically necessary; within the class, however, the choice is not neutral, and a regimen with lower QT liability and less CYP3A4 dependence is preferable in a patient receiving amiodarone. Domain 4 is amber: monitoring is achievable, but only in a supervised setting. Under the precedence rules, two red domains including Domain 1 resolve to monitored initiation after multidisciplinary review rather than to deferral: dabrafenib and trametinib are started in a supervised setting, guideline-directed HF therapy is continued with dose adjustment, and daily weight, electrolytes and ECG are recorded, with echocardiography at baseline and at two and four weeks and predefined escalation criteria. At four weeks an asymptomatic fall in LVEF from 42% to 36% meets the IC-OS definition of moderate CTRCD. This triggers re-grading rather than automatic permanent discontinuation: treatment is temporarily interrupted, HF therapy is uptitrated, LVEF recovers to 44%, and treatment is reintroduced at a reduced dose with four-weekly imaging—a sequence consistent with the reversibility reported in real-world and prospective cohorts [9,13].

#### 3.7.3. Case 3: Suspected ICI Myocarditis on a Background of Chronic Heart Failure

Three weeks after the first dose of combination checkpoint blockade, the patient described in Case 1 reports increased fatigue and mild exertional dyspnea—symptoms he has had for years. High-sensitivity troponin T has risen from a baseline of 22 ng/L to 180 ng/L, the ECG shows new first-degree atrioventricular block with right bundle branch block, LVEF is unchanged at 37%, and NT-proBNP has risen from 900 to 1400 pg/mL. This is the situation for which the framework was designed: the symptoms are non-discriminating, the ejection fraction is uninformative because it was already reduced, and the decisive findings are the change from a documented baseline and the new conduction abnormality. Checkpoint blockade is withheld immediately, the patient is admitted for telemetry, and intravenous methylprednisolone 1000 mg daily is started within 24 h without waiting for cardiac magnetic resonance, while acute coronary syndrome, pulmonary embolism, infection and progression are excluded and creatine kinase, proximal and respiratory muscle strength, ptosis, diplopia and dysphagia are assessed for myositis or myasthenic overlap [3,20,45,49]. Two HF-specific actions are planned simultaneously: diuretic dose is increased pre-emptively with daily weights and daily potassium and glucose monitoring, because high-dose corticosteroid therapy is a predictable congestive stressor in HFrEF; and infliximab is excluded from the escalation plan in advance, with abatacept-based or mycophenolate-based options identified instead should the patient prove refractory [46,47,50,51]. On re-grading, Domain 1 and Domain 3 are now both red for checkpoint blockade; because Domain 2 remains amber and the tumor is BRAF V600E-mutated, the framework directs the team to a change in class rather than to rechallenge.

#### 3.7.4. Case 4: HFpEF, Frailty and Limited Monitorability

An 82-year-old woman has HFpEF (LVEF 58%) with permanent atrial fibrillation, chronic kidney disease stage 3b (eGFR 34 mL/min/1.73 m^2^), obesity and diuretic-dependent congestion. Her G8 screening score is 11, she lives 120 km from the treating center, has no caregiver able to support frequent visits, and states that maintaining independence matters more to her than maximal treatment intensity. Her melanoma is BRAF wild-type; so, checkpoint blockade is the only effective systemic option. Domain 1 is amber, with the explicit caution that a preserved ejection fraction must not be read as low risk in a patient who is congestion-prone with atrial fibrillation and renal impairment. Domain 2 is amber. Domain 3 is amber to red: myocarditis is rare, but conduction disease and arrhythmia would be poorly tolerated, and immune-mediated colitis in a diuretic-dependent patient with chronic kidney disease carries a high risk of hypovolemia and acute kidney injury. Domain 4 is red, driven by distance, absent support, frailty and stated priorities. Under the fourth precedence rule the plan, not the patient, is downgraded: single-agent anti-PD-1 rather than combination therapy, formal comprehensive geriatric assessment prompted by the G8 score [28,29], shared care with the local hospital for ECG and troponin, written sick-day rules for diuretics and anticoagulation, a direct telephone route with a symptom-triggered pathway, and an explicit goals-of-care record. Inability to attend intensive surveillance is treated as a reason to restructure the monitoring plan, not as a reason to withhold effective therapy.

## 4. Discussion

### 4.1. What an HF-Centered Review Adds Beyond General Cardio-Oncology Guidance

ESC cardio-oncology guidance, IC-OS definitions, and prior JACC: CardioOncology reviews provide the essential general framework for cardiovascular toxicity [3,22,36]. Repeating those recommendations without changing the decision problem would add little. The present review therefore centers on five consequences of HF already being present: baseline symptoms and biomarkers are often abnormal; ventricular reserve differs across HF phenotypes; decompensation may require stabilization before treatment; standard interruption thresholds must be interpreted against the patient’s trajectory and oncological urgency; and polypharmacy, renal dysfunction, frailty, and device dependence can alter both toxicity and monitorability.

Because the limitations of these sources are what motivate the present framework, they should be stated specifically rather than in general terms. The 2022 ESC cardio-oncology guidelines provide the structure for baseline assessment and surveillance, but their recommendations for BRAF/MEK inhibitors and ICIs rest largely on expert consensus rather than randomized evidence, and the HFA-ICOS proformas were derived by consensus and have not been prospectively validated in melanoma [3,26]. Within the HFA-ICOS “RAF/MEK inhibitors” proforma, which covers the BRAF/MEK inhibitor combinations used in melanoma, “previous heart failure or cardiomyopathy” is a single very-high-risk item that is not graded by ejection-fraction phenotype, current stability, or time since decompensation, and no management pathway follows from the classification. The discrimination of the tool in melanoma is also endpoint-dependent: in one retrospective cohort 82% of CTRCD occurred in patients classified as low or medium risk [10], whereas in the prospective cohort no low-risk participant developed moderate or severe CTRCD although 37.9% developed mild CTRCD [13]. Surveillance intervals are pragmatic rather than evidence-derived: echocardiography approximately every four months is suggested for high or very-high-risk patients, whereas the prospective data indicate that the decisive window is four weeks. Most importantly for this review, the guidance assumes an interpretable baseline. It does not specify how to define an event when ejection fraction, strain and natriuretic peptides are already abnormal; it does not address what should be done when the recommended period of cardiovascular optimization is itself oncologically unsafe; and it offers no dedicated recommendations for HFpEF, predominant right ventricular failure, device dependence, or advanced HF in this setting.

The IC-OS statement provides an indispensable severity vocabulary, but three features limit its application here [22]. Its thresholds were developed and are best supported in anthracycline- and trastuzumab-treated populations. The mild CTRCD category depends on a relative fall in global longitudinal strain, which requires a vendor-consistent, high-quality baseline that is frequently unobtainable in patients with atrial fibrillation, poor acoustic windows, or established remodeling. The biomarker criterion is poorly operationalized when troponin and natriuretic peptides are chronically elevated, and the fallback rule of a further 20% rise is pragmatic rather than validated in HF. Two further problems arise in practice: the definitions were designed to classify severity rather than to dictate management, yet they are commonly applied as interruption thresholds; and a single severity category can be satisfied by three different processes—progression of the underlying cardiomyopathy, treatment-associated ventricular dysfunction, and myocarditis—that demand different responses. The symptomatic categories are additionally weakened in this population because they rest on NYHA class, which is already abnormal before therapy begins.

Prior JACC: CardioOncology reviews, including the state-of-the-art review of BRAF and MEK inhibitor cardiovascular toxicity [36], provide the most complete mechanistic and class-level synthesis available, but they are organized by drug class rather than by patient substrate; so, the implicit index patient has a structurally normal heart. They describe the detection and management of incident toxicity rather than decision-making when the cardiac abnormality precedes therapy; they do not condition sequencing on cardiac status, address competing oncological urgency, or provide rechallenge guidance in chronic HF; and the evidence they synthesize derives predominantly from trial populations from which clinically significant cardiovascular disease was excluded; so, the estimates are not transportable to an HF cohort. Several also predate the first prospective cardiovascular cohort study in this setting [13].

Identifying these limitations does not resolve them. The framework proposed here adds no new evidence; what it adds is an explicit decision structure that states where the existing guidance stops, which variable is being weighed against which, and what should be recorded when the answer is uncertain.

The proposed four-domain framework is the conceptual contribution of this review. It links HF substrate and stability with tumor urgency, class-specific toxicity, and detectability/reversibility rather than treating “pre-existing HF” as a single high-risk label. It also distinguishes three different clinical events that are often conflated: progression of the patient’s underlying HF, treatment-associated ventricular dysfunction, and ICI-mediated myocarditis. These events require different attribution, monitoring, interruption, and rechallenge strategies. The framework remains hypothesis-generating because direct HF-specific comparative evidence is absent.

### 4.2. Applying the Four-Domain Framework to a Clinical Case

The practical value of the framework is best demonstrated by a case in which guideline text alone does not produce an answer. Consider the composite patient described in Case 2 (Section 3.7): a 59-year-old woman discharged twelve days earlier after a HF admission, with HFmrEF (LVEF 42%), permanent atrial fibrillation on amiodarone and apixaban, moderate mitral regurgitation and an NT-proBNP falling from 4800 to 2600 pg/mL on a diuretic regimen that has not yet stabilized, who presents with BRAF V600E-mutated melanoma, symptomatic liver metastases, a rising lactate dehydrogenase and performance status 2.

Read through existing guidance alone, this patient is classified as very high risk before combined BRAF/MEK inhibition and would ordinarily be advised to undergo cardiovascular optimization before treatment [3,26]. The guidance does not state what to do when that period of optimization is the interval during which the melanoma is expected to become untreatable. The framework requires both considerations to be graded rather than one of them to be assumed. Domain 1 is red because of decompensation within 30 days and an unstable diuretic requirement. Domain 2 is also red because progression is rapid, organ function is threatened, and immunotherapy-first sequencing—although supported at population level [37]—has a slower expected time to response than this patient can tolerate. Domain 3 is red for the class that is oncologically necessary, since ventricular dysfunction, hypertension and QT prolongation are the toxicities this substrate tolerates least; within the class the choice is nevertheless not neutral, and a pair with lower QT liability and less CYP3A4 dependence is preferable in a patient receiving amiodarone. Domain 4 is amber: monitoring is deliverable, but only in a supervised setting rather than a standard outpatient pathway.

The composite pattern—three red domains, one of which is Domain 1—resolves under the precedence rules neither to “do not treat” nor to “treat as usual”, but to monitored initiation after multidisciplinary review. In practice this means dabrafenib and trametinib started at full dose in a supervised setting, continued guideline-directed HF therapy with dose adjustment, daily weight and electrolytes, echocardiography at baseline and at two and four weeks, predefined escalation and interruption criteria, and an explicit written record of why delay was judged unsafe. At four weeks an asymptomatic fall in LVEF from 42% to 36% satisfies the IC-OS definition of moderate CTRCD. Under the framework this triggers re-grading rather than automatic permanent discontinuation, and the documented response—temporary interruption, uptitration of HF therapy, recovery to 44%, and reintroduction at a reduced dose with four-weekly imaging—is consistent with the reversibility reported in both real-world and prospective cohorts [9,13].

The purpose of the exercise is not to claim that this sequence is proven correct; no comparative evidence exists, and a different multidisciplinary team could reasonably have decided otherwise. The point is that the same case processed through the framework yields a decision that is explicit, auditable and revisable, together with a record of the trade-off that was accepted and the criteria that would reverse it. That is precisely what general cardio-oncology guidance leaves undefined when it is applied to a patient whose cardiac baseline is already abnormal and whose cancer will not wait.

### 4.3. Epistemic Status of the Framework and the Separation of Guideline-Supported from Author-Proposed Statements

Because the framework proposed here is an expert construction applied to a population for which the direct evidence is case-level, the risk that it will be read as guidance is real, and it is better mitigated by explicit mapping than by a general disclaimer. Table S2 therefore sets each principal recommendation of this review against its source, states what that source actually says, and states what the present review adds. Three areas deserve particular emphasis because they are the ones most likely to be misread.

The first is early imaging reassessment. Current guidance suggests echocardiography approximately every four months in patients at high or very high risk [3]; the reassessment at approximately four weeks that we propose for unstable, recently decompensated or very-high-risk patients appears in no guideline and is extrapolated from a single prospective observational cohort of 61 analyzed patients in which pre-existing left ventricular systolic dysfunction was present in one participant [13]. It is a defensible risk-adapted choice, not a standard of care, and a team that images at three months is not deviating from guidance. The second is treatment interruption and rechallenge. IC-OS provides a severity vocabulary, not interruption thresholds [22], and the criteria we propose for patients whose baseline is already abnormal—change from the personal reference value, trajectory on repeat imaging, and explicit attribution among progression of the underlying HF, treatment-associated dysfunction and myocarditis—are author-derived and are offered as a documentation standard rather than as evidence about safety. The third is phenotype-specific management. No current guideline provides recommendations for HFpEF, predominant right ventricular failure, device dependence or advanced HF in the context of systemic melanoma therapy; so, everything in Table 4 and in Section 3.1.4 is an author proposal that happens to be consistent with general HF guidance rather than an application of cardio-oncology guidance.

Two consequences follow. Where a guideline recommendation and a proposal made here diverge, the guideline recommendation should prevail, and the divergence should be recorded rather than silently resolved. And where an author-proposed element is adopted locally—for example a four-week echocardiogram in a very-high-risk patient—it should be documented as a local risk-adapted decision, which is also the form in which it could later be audited or tested prospectively.

A framework proposed within a review that emphasizes how limited the evidence is invites an obvious objection: either the evidence supports a structured approach, in which case the pessimism is overstated, or it does not, in which case the structure is decoration. We think the objection dissolves once the claim being made is stated precisely, and it must be stated precisely, because allowing a reader to assume that a systematically presented framework was systematically derived would be the most damaging misreading of this article.

The framework makes a procedural claim rather than an empirical one. It asserts that four questions determine what happens to a patient with pre-existing HF who requires systemic melanoma therapy; that these questions are at present usually answered implicitly, inconsistently and without a record; and that answering them explicitly produces documentation that can be taught, audited and revised. It asserts nothing whatever about the magnitude of any risk, the comparative safety of any regimen, or the probability of any outcome. Those are empirical claims, and the evidence summarized in Table 1 and Table 5 cannot support them; that is the point of those tables. Nothing in the framework was derived from a dataset, weighted from outcomes, or validated prospectively, and the descriptors in Table 2 and Table 3 are conventions rather than cut-points. Two multidisciplinary teams applying the framework faithfully to the same patient may reasonably reach different decisions. If both records show which variable was weighed against which, what was accepted, and what would reverse the judgement, the framework has done the whole of the work it claims to do.

Stated negatively, which is the more useful direction here, the framework does not convert weak evidence into recommendations, does not confer authority on the authors’ preferences, and does not license a decision that a clinician would otherwise have judged unsafe. It also does not resolve the underlying problem: only prospective data stratified by HF phenotype and stability can do that, and Section 4.6 sets out what such data would have to report. Table 10 states these limits in the form of what the framework does and does not do. We would regard the framework as having failed if it were cited as a validated instrument, and as having succeeded if it were used to structure a multidisciplinary record and then superseded by evidence.

### 4.4. Heart Failure with Preserved Ejection Fraction as a Distinct Decision Context

Section 3.1.4 sets out why a preserved ejection fraction cannot serve as the change variable during melanoma therapy. The wider point for cardio-oncology is that HFpEF is a systemic rather than a purely cardiac phenotype: adiposity, insulin resistance, adipose-tissue inflammation, neurohormonal and sodium-handling abnormalities, and impaired renal reserve generate the substrate and continue to operate throughout cancer treatment, and the same pathways also connect obesity with cachexia and sarcopenia at the other end of the nutritional spectrum [52]. Four practical corollaries follow for the patient receiving systemic melanoma therapy. Natriuretic peptides are lower for any given filling pressure in obesity; so, an apparently reassuring value is less reassuring than in a lean patient and the trajectory matters more than the absolute number. Body weight, which is the most useful single congestion marker in HF, becomes ambiguous during cancer therapy because treatment-associated anorexia, sarcopenia and cachexia move it in the opposite direction to fluid accumulation; so, weight must be interpreted together with clinical congestion rather than alone. The metabolic substrate that produces HFpEF also predicts the complications of the treatment given for immune-related toxicity, since high-dose corticosteroids will more readily precipitate hyperglycemia, sodium retention and congestion in precisely these patients. And functional capacity, rather than ejection fraction, is the variable that both the patient and the oncologist are actually trying to protect, which argues for recording an objective measure of it at baseline.

This has a direct implication for the evidence base. Because HFpEF deterioration is invisible to an ejection-fraction endpoint, the existing cohorts cannot have detected it: a patient who developed congestion and required hospitalization with an unchanged LVEF would not have met the CTRCD definition used in any of the studies summarized in Table 5. The absence of reported HFpEF events in those cohorts should therefore not be read as evidence of safety in this phenotype. Prospective registries in this field should report HFpEF as a separate stratum and should include congestion, diuretic requirement, natriuretic-peptide trajectory, renal function, atrial fibrillation burden and functional capacity among their outcomes, or the phenotype will remain invisible for a second generation of studies.

### 4.5. Competing Cardiovascular and Oncological Risk

In advanced HF, in frailty, and in active or recently decompensated HF, the decision is no longer only whether a treatment is cardiotoxic. It is whether the anticipated oncological benefit outweighs both the cardiovascular vulnerability and the competing risk of death from HF itself, and this is a different question that the cardiotoxicity literature does not answer. The magnitude of the competing risk is not negligible: mortality in the year following an HF hospitalization remains of the order of one in five, and it is substantially higher in advanced HF, in inotrope dependence and in the presence of significant renal dysfunction [23]. Where that horizon is shorter than the time in which a melanoma treatment could deliver meaningful benefit, an intensive treatment and surveillance strategy may impose burden without altering outcome.

We suggest that four questions be answered explicitly and recorded before starting, and again at every re-grading (author-proposed). What is the expected oncological benefit, and over what time horizon? What is the expected time to response of each feasible option, given that this is the variable that determines how much cardiovascular instability must be tolerated rather than corrected? What is the patient’s competing cardiovascular and non-cancer mortality, and does it fall inside or outside that horizon? And would the result of a proposed surveillance test actually change management, given the treatment options that remain acceptable to this patient? A test whose result cannot change the plan is a burden rather than a safeguard, and this is the point at which protocolized imaging should give way to a symptom-triggered pathway.

The answers move the plan in both directions rather than only towards de-escalation. When competing cardiovascular mortality dominates, treatment intensity and surveillance intensity should fall together and coherently—single-agent rather than combination checkpoint blockade, symptom-triggered rather than scheduled imaging, an explicit goals-of-care record—because reducing one while maintaining the other produces the worst combination of burden and benefit. When the melanoma is the dominant threat, cardiovascular optimization must not consume time the patient does not have, which is the reasoning behind the second precedence rule and behind the requirement that the record state why delay was judged unsafe. Frailty modifies both sides of the balance simultaneously, reducing physiological reserve while also shortening the horizon over which benefit can accrue, and it is for this reason that a brief geriatric screen is treated in this framework as part of the oncological assessment rather than as an optional addition [28,29]. Domains 2 and 4 encode this asymmetry, but the judgement itself cannot be automated; what the framework requires is that it be made explicitly and written down, because it is the judgement most likely to be questioned later.

### 4.6. Evidence Gaps and Limitations of Current Data

The principal limitation is not merely that HF patients are “high risk,” but that the evidence cannot quantify how much risk changes according to HF phenotype or stability. Pivotal trials commonly excluded clinically significant cardiovascular disease, while real-world studies rarely report dedicated strata for HFrEF, HFmrEF, HFpEF, recent decompensation, right-sided HF, advanced HF, or device therapy. Consequently, this review cannot support a therapy-specific comparative risk estimate in pre-existing HF and should not imply that observational incidence in unselected cohorts applies directly to this population.

Cross-study comparison is further limited by evolving CTRCD definitions, different imaging schedules, variable use of GLS and biomarkers, and incomplete adjudication of competing causes [22]. HFA-ICOS provides a practical therapy-specific framework but has not been prospectively validated for melanoma patients with HF, and low- or medium-risk classifications do not eliminate risk [10,26].

Pharmacovigilance databases identify signals but cannot determine incidence because reporting is selective, duplicate reports may occur, and denominator and baseline cardiac data are absent [12,21]. Likewise, ICI myocarditis estimates depend strongly on surveillance and case definition. The present review is additionally limited by its narrative design: article selection and interpretation may be influenced by our judgment, no formal study-level risk-of-bias or GRADE assessment was undertaken, and no quantitative synthesis was possible. Dedicated prospective registries should enroll patients across HF phenotypes and geriatric vulnerability levels, document baseline stability and guideline-directed therapy, apply standardized IC-OS criteria, and report treatment continuation, recovery, cancer outcomes, function, and quality of life.

### 4.7. SGLT2 Inhibitors: A Hypothesis Rather than an Established Cardioprotective Strategy

SGLT2 inhibitors reduce HF hospitalization across the ejection-fraction spectrum and should be continued, or started, whenever they are independently indicated for HF [24,25]. There is, however, no evidence that they protect against BRAF/MEK inhibitor-associated CTRCD or against ICI myocarditis. The available support consists of mechanistic extrapolation and a single case in which cardiac recovery followed drug withdrawal together with multidrug HF therapy that happened to include an SGLT2 inhibitor [8], an observation that cannot separate the contribution of any one component. This is therefore a hypothesis rather than a cardioprotective strategy, and it is presented as such: in this setting SGLT2 inhibition is indicated HF therapy, not prophylaxis against a specific cancer-therapy toxicity, and the practical priorities during melanoma treatment are the sick-day rules and the monitoring for euglycemic ketoacidosis set out in Table 6. Whether these agents prevent CTRCD or accelerate recovery from it is a question for prospective study and should not be assumed in the interim.

### 4.8. Sequencing of Therapies in BRAF-Mutated Melanoma with Cardiac Comorbidity

DREAMseq showed an overall survival advantage for an immunotherapy-first sequence in eligible patients with advanced BRAF-mutated melanoma [37]. This population-level oncological result is important but does not create a universal sequence for patients with unstable HF, prior myocarditis, severe conduction disease, markedly reduced LVEF, or urgent need for rapid tumor response. Cardiac status, tumor burden and tempo, prior therapy, expected time to response, and availability of alternatives should be weighed together.

The classes pose different clinical problems: BRAF/MEK inhibitors more often cause ventricular dysfunction that may be asymptomatic and reversible, whereas ICI myocarditis is rarer but can progress rapidly with conduction, arrhythmic, and neuromuscular complications. It is not valid to assume that a patient with severely reduced LVEF necessarily “tolerates” one class better than the other. Sequencing must remain individualized because comparative cardiovascular safety data in established HF do not exist.

### 4.9. Multidisciplinary Team: Structure and Function

A practical multidisciplinary team includes medical oncology, cardiology/cardio-oncology, clinical pharmacy or pharmacology, and specialist nursing, with neurology, intensive care, electrophysiology, and advanced HF expertise added when indicated. The team should document baseline risk, the evidentiary uncertainty, the selected regimen, a monitoring plan, thresholds for urgent review, and the rationale for continuation, interruption, dose modification, discontinuation, or rechallenge. Clear documentation improves continuity without implying that every decision requires a formal meeting.

### 4.10. Older and Frail Patients

Older and frail adults represent a clinically important proportion of the advanced-melanoma population but remain less well represented in pivotal trials than younger, fitter patients. Population-based and real-world studies help address this external-validity gap. In older patients treated outside trials, ICIs can retain clinical effectiveness, while BRAF/MEK-targeted therapy also remains active; however, toxicity, dose reduction, and treatment discontinuation are frequent and are not captured adequately by chronological age alone [4,27].

Frailty, functional status, cognition, nutrition, renal function, multimorbidity, polypharmacy, falls, social support, and treatment goals should therefore complement cardiovascular assessment. A brief geriatric screen such as G8 can identify patients who may benefit from comprehensive geriatric assessment and targeted interventions. Observational data suggest that frailty may be associated with greater toxicity-related hospitalization or discontinuation, although estimates remain imprecise [28]. SIOG recommendations emphasize individualized toxicity recognition and management in older adults receiving ICIs [29].

In a patient with both HF and geriatric vulnerability, treatment planning should avoid undertreatment based on age alone while recognizing lower physiological reserve, competing mortality, orthostatic risk, renal dysfunction, sarcopenia, and the functional consequences of even low-grade toxicity. Monitoring schedules should be feasible, caregiver-supported when necessary, and linked to clear symptom-triggered pathways. Future melanoma trials should deliberately include older and frail patients and report geriatric, cardiovascular, functional, and patient-reported outcomes.

Beyond the general principle that chronological age is a poor proxy for physiological reserve, the decision in an older patient with metastatic melanoma and pre-existing HF turns on a small number of concrete questions that are seldom recorded and cannot be inferred from performance status, ejection fraction or a frailty score. Six recur often enough to be asked systematically, and they are set out with their consequences in Table 11.

The first is the weight the patient places on overall survival relative to other endpoints. Melanoma therapy is now capable of long survival in a subgroup, and this properly dominates the discussion in a fit patient; in an older patient with HF it may not, and some will prefer a more predictable course to a higher expected survival obtained through a more toxic sequence. The second is quality of life, which becomes operational only when the patient names the symptoms or limitations he or she would least tolerate, because those are the toxicities that should trigger dose modification before they reach a formal severity grade. The third is the avoidance of hospitalization. This is an endpoint in its own right for many older patients, and it is also the endpoint on which the two treatment classes differ in mechanism rather than in magnitude: admissions during BRAF/MEK inhibition arise mainly from hypertension, congestion, and acute kidney injury following gastrointestinal toxicity, whereas admissions during checkpoint blockade arise from immune-related events and from the congestive and metabolic consequences of the corticosteroids used to treat them.

The fourth is the willingness, as distinct from the ability, to accept frequent monitoring. The surveillance schedules proposed in Table 7 are demanding in the first twelve weeks, and a patient who cannot or will not attend weekly during the first month is not a candidate for a strategy that depends on it. This is a reason to restructure the monitoring plan under the fourth precedence rule—shared care with a local hospital, a written symptom-triggered pathway, a direct telephone route—and not a reason to withhold effective therapy. The fifth is the preference between an oral and an infusion regimen, which is a genuine clinical variable rather than a matter of convenience wherever both classes are oncologically reasonable. Oral BRAF/MEK inhibition removes the travel burden but transfers responsibility to the patient for daily adherence, home blood-pressure measurement, electrolyte and interaction discipline, and the recognition of continuous low-grade toxicity; infusion checkpoint blockade removes the daily burden and the adherence risk but requires attendance at the center, which in a patient living at a distance and without a caregiver may be the harder of the two. Cognitive impairment, polypharmacy and the absence of support therefore push in opposite directions in different patients, and the choice should be made with the patient rather than assumed from the diagnosis. The sixth is the functional expectation: the level of independence or mobility below which the patient would not wish treatment to continue, stated concretely and in advance; so, that it can be applied if it is reached rather than improvised during a crisis.

These preferences are not a soft appendix to the cardiological assessment. They determine which of the strategies generated by Domains 1 to 3 can actually be delivered, and they frequently determine the answer where the cardiological and oncological considerations are finely balanced. They should be established before the first exposure, recorded in the patient’s own words in Domain 4, and revisited after any hospitalization, any significant toxicity and any change in disease status, because a patient who has experienced an immune-related complication or an HF admission often answers these questions differently afterwards. Where a stated priority conflicts with the surveillance strategy the framework would otherwise propose, the plan is downgraded rather than the patient, and the conflict itself is recorded, since it is the element of the decision least likely to be reconstructable later.

## 5. Conclusions

The clinically relevant question is not whether systemic melanoma therapies are cardiotoxic in general, but how treatment should be adapted when HF is already present. Direct evidence remains case-level and most incidence estimates are indirect, and three messages follow from that position.

First, pre-existing HF is not an automatic contraindication to effective melanoma therapy. Stable HF should be treated as a determinant of how a patient is monitored rather than as a reason for exclusion, whereas recent or active decompensation warrants stabilization or initiation in a monitored setting when oncological delay would itself be unsafe.

Second, HF phenotype and current stability, rather than the diagnostic label, should govern both treatment selection and surveillance. An abnormal baseline must be documented and then used as the patient’s own reference value, a preserved ejection fraction must not be read as low risk, and the four-domain framework with its precedence rules is offered as an explicit and auditable way of recording that reasoning, with every recommendation identified as guideline-supported or author-proposed. The framework is a documentation structure and not a decision algorithm: it does not convert limited evidence into recommendations, it has not been validated, and in older patients, it is incomplete until the patient’s own priorities—survival, quality of life, avoidance of hospitalization, tolerance of monitoring, route of administration and functional expectation—have been recorded alongside the cardiological data.

Third, prospective HF-specific cardio-oncology data are urgently required. Registries and trials should deliberately include stable HF across the ejection-fraction spectrum and report phenotype, congestion, right ventricular and valvular disease, devices, biomarkers, HF therapy, interruption, recovery, rechallenge, cancer outcomes, function and quality of life. Until those data exist, the framework proposed here should be used as a structure for documented clinical reasoning and not as a validated risk score.

## Figures and Tables

**Table 1:** Applicability of the principal evidence streams to melanoma patients with pre-existing heart failure.

Evidence Source	Population and Baseline Cardiac Status	Key Observation	Applicability to Established HF
Nishizawa et al. [7]	Single patient with BRAF V600-mutated melanoma and NYHA class III HF; HF may have been partly driven by malignant effusions/hemodynamic effects.	ICI therapy followed by encorafenib/binimetinib achieved tumor response without major treatment-induced cardiac toxicity; HF improved.	Direct but anecdotal. Demonstrates feasibility in one carefully selected patient; cannot estimate incidence, comparative safety, or optimal sequencing.
Benko et al. [8]	Single patient without documented pre-existing HF who developed BRAF/MEK inhibitor-associated CTRCD.	Cardiac phenotype and function recovered after treatment withdrawal and HF therapy, including an SGLT2 inhibitor.	Indirect. Supports potential reversibility but does not establish prevention or treatment efficacy in patients with pre-existing HF.
Real-world BRAF/MEK cohorts [9,10,11,12,13]	Unselected melanoma cohorts; established HF was uncommon, excluded, or not reported as a dedicated outcome stratum.	Reported cardiac dysfunction/CTRCD varied from approximately 18% to 27% according to definitions and monitoring intensity.	Indirect. These are not HF-specific incidence estimates and should not be applied quantitatively to patients with established HF.
ICI myocarditis cohorts and pharmacovigilance studies [14,15,16,17,18,19,20,21]	Mixed cancer populations or unselected ICI-treated cohorts; no validated melanoma-with-HF subgroup analysis.	Myocarditis is uncommon but may be severe, early, and associated with conduction disease, arrhythmia, and neuromuscular overlap.	Indirect. Diagnostic and treatment principles are extrapolated; baseline HF-specific risk remains uncertain.
ESC, IC-OS, HFA-ICOS and HF guidance [3,22,23,24,25,26]	Cross-cancer consensus, therapy-specific risk tools, and general HF trial populations.	Provides definitions, baseline assessment, surveillance concepts, and guideline-directed HF therapy.	Extrapolated. Useful for structured care, but not prospectively validated specifically in melanoma patients with established HF.
Older/frail melanoma cohorts and geriatric-oncology guidance [4,27,28,29]	Older adults treated with ICIs or targeted therapy in routine practice; frailty and HF were inconsistently measured, and dedicated melanoma-with-HF strata were unavailable.	Real-world data indicate treatment activity in older patients but also clinically relevant toxicity, dose modification, hospitalization, and discontinuation. Frailty may be more informative than chronological age alone.	Indirect. Supports geriatric screening and individualized care, but does not quantify cardiotoxicity in older or frail patients with established HF.

**Table 2:** Ordinal grading of the four domains and the specific decision output produced by each domain. The green/amber/red descriptors, numerical or temporal cut-points, precedence logic, and decision outputs are author-proposed (AP), have not been prospectively validated, and must not be interpreted as a risk score. References inserted in the descriptor cells support the underlying heart-failure, cardio-oncology, melanoma, treatment-toxicity, pharmacologic, and geriatric concepts; they do not validate the proposed ordinal thresholds.

Domain	Green	Amber	Red	Decision Output Produced by This Domain
Domain 1 HF substrate and current stability	Chronic stable HF or HF with improved ejection fraction; no congestion; unchanged diuretic dose for ≥3 months; no HF hospitalization in the past 12 months; stable renal function; tolerating guideline-directed therapy [3,22,23,26].	HFrEF or HFmrEF with LVEF 35–49%, or congestion-prone HFpEF; chronic atrial fibrillation; moderate valvular disease; CKD stage 3; chronically abnormal but stable natriuretic peptides or troponin; device in situ without recent therapies [3,22,23,26].	HF hospitalization or decompensation within 30 days; escalating diuretic requirement; symptomatic hypotension; symptomatic LVEF <35%; severe untreated valvular disease; uncontrolled ventricular arrhythmia or recent ICD therapies; advanced HF or inotrope dependence [3,22,23,26].	Timing of therapy: proceed now, optimize first, or treat only in a monitored setting. Defines the mandatory baseline dataset (symptoms, blood pressure, ECG, LVEF and GLS where feasible, RV and valve assessment, troponin, BNP/NT-proBNP, renal function, electrolytes, device interrogation). [AP]
Domain 2 Oncological urgency and alternatives	Adjuvant setting or low-burden disease; effective alternatives available; delay of several weeks acceptable without measurable harm [1,2,37].	Advanced disease with meaningful burden but no threatened organ function; delay of 1–2 weeks acceptable; an alternative class remains feasible [1,2,37].	Rapid progression, symptomatic visceral or central nervous system disease, rising LDH, threatened organ function; no effective alternative; delay likely to be harmful within days [1,2,37].	Acceptable delay window in days or weeks; whether a locoregional, alternative systemic, or best-supportive-care option exists; explicit statement of the harm expected from delay and of the trade-off accepted.
Domain 3 Treatment-specific toxicity phenotype	The dominant toxicity phenotype of the planned regimen does not overlap the principal vulnerability (for example anti-PD-1 monotherapy in HFpEF without conduction disease or prior myocarditis) [3,22,26,36].	Partial and mitigable overlap (for example BRAF/MEK inhibition with baseline hypertension; combination checkpoint blockade in a patient with prior atrial arrhythmia; moderate QT prolongation correctable by electrolyte management) [3,22,26,30,31,32,33,34,35,36,38,39,40].	Direct overlap (for example BRAF/MEK inhibition in symptomatic HFrEF with LVEF <40%; a QT-prolonging regimen with QTc >480 ms, concomitant amiodarone, or an unavoidable strong CYP3A4 interaction; checkpoint blockade in pacing-dependent or high-grade conduction disease, or after previous myocarditis) [3,22,26,30,31,32,33,34,35,36,38,39,40].	Regimen and sequence; surveillance content and timing (blood pressure, ECG, electrolytes and serial ventricular function for BRAF/MEK inhibitors; early symptom review, ECG and troponin for ICIs); management category assigned to every concomitant cardiovascular medicine.
Domain 4 Detectability, reversibility and patient priorities	Interpretable baseline; echocardiography and troponin obtainable within 72 h; reliable attendance; caregiver support available; goals aligned with active anticancer treatment [3,22,26,28,29].	Baseline partly uninterpretable (poor acoustic windows, atrial fibrillation, strain unavailable); intermittent access to imaging or biomarkers; possible frailty (G8 ≤14) not yet formally assessed [3,22,26,28,29].	No timely access to imaging or biomarkers; inability to attend early reassessment; significant cognitive impairment or absent support; patient priorities favoring symptom control over intensive surveillance; a severe toxicity could not be managed in the available setting [3,22,26,28,29].	Whether the plan is deliverable and where care should occur; written symptom-triggered pathway; patient-specific interruption, resumption and rechallenge criteria; documented goals-of-care discussion.

**Table 4:** HF phenotype-specific implications for systemic melanoma therapy. All entries in this table are author-proposed (AP) risk-adapted syntheses: current cardio-oncology guidance does not provide phenotype-specific recommendations for this setting, and no comparative melanoma data exist across HF phenotypes.

HF Substrate	Baseline Interpretation Issue	Implication for Therapy Selection/Readiness	Monitoring and Decision Points
Stable HFrEF or HFmrEF	Reduced LVEF and abnormal BNP/NT-proBNP may be chronic; small absolute changes may be clinically important when reserve is limited.	Optimize indicated HF therapy and volume status. No automatic exclusion, but use treatment-specific HFA-ICOS assessment and document acceptable LVEF/GLS and symptom thresholds.	Early clinical review; serial BP, ECG and ventricular function according to regimen and baseline risk. Compare biomarkers and imaging with the patient-specific baseline.
Stable HFpEF	LVEF may remain normal despite congestion, pulmonary hypertension, atrial fibrillation, or impaired reserve.	Assess filling pressures clinically, BP, rhythm, renal function, obesity/diabetes and diuretic needs. Avoid interpreting preserved LVEF as low risk.	Track weight, congestion, BP, renal function and natriuretic-peptide trajectory; investigate new dyspnea beyond LVEF alone.
Recent or active decompensated HF	Symptoms and biomarkers are unstable, making attribution of treatment toxicity unreliable.	Stabilize before non-emergent therapy. If cancer delay is unsafe, consider monitored initiation with oncology, cardio-oncology and acute-HF support.	Frequent reassessment, explicit escalation criteria and rapid access to imaging/biomarkers; avoid outpatient-only pathways when instability persists.
Predominant RV failure, pulmonary hypertension, or severe valve disease	Standard LVEF surveillance may miss the principal hemodynamic limitation.	Define RV function, pulmonary pressures and valve severity before treatment; assess whether tumor burden, effusion or pulmonary vascular disease contributes.	Include RV and valve parameters, volume status, oxygenation and rhythm; escalate unexplained hypotension, edema or hypoxemia promptly.
Advanced HF, devices, or recurrent ventricular arrhythmia	Symptoms, pacing, ICD therapies and chronic biomarker abnormalities complicate routine algorithms.	Use advanced-HF/electrophysiology input. Consider treatment setting, competing prognosis, device interactions and patient goals.	Device interrogation when indicated, electrolyte/QT surveillance, documented resuscitation and treatment-interruption plans, and individualized goals-of-care discussion.

**Table 5:** Design, population, regimen, surveillance, follow-up duration and event definitions of representative sources reporting BRAF/MEK inhibitor-associated cardiovascular toxicity in melanoma. Values in different rows are not directly comparable and none is HF-specific; the table is presented in place of a graphical comparison precisely because the defining characteristics, rather than the headline percentages, explain the differences between studies. CTRCD, cancer therapy-related cardiac dysfunction; GLS, global longitudinal strain; LVEF, left ventricular ejection fraction; MUGA, multigated acquisition scan; pp, percentage points.

Source, Design, Setting and Period	Sample Size and Population	Regimen(s)	Cardiac Surveillance and Follow-Up Duration	Event Definition	Reported Cardiac Outcome and Applicability to Established HF
Oddershede et al. [9] Retrospective real-world cohort; two Danish university hospitals; 2015–2023.	*n* = 170 patients with metastatic melanoma treated in routine care.	Encorafenib + binimetinib, vemurafenib + cobimetinib, or dabrafenib + trametinib.	Echocardiography or MUGA at baseline and every 3 months throughout treatment. Mean time to LVEF decline 187 days; 92% of major events occurred within the first year.	Major cardiotoxicity: LVEF fall ≥10 pp to LVEF < 50%. Minor cardiotoxicity: LVEF fall ≥15 pp with LVEF remaining >50%. Imaging only; no strain or biomarker criterion.	Any cardiotoxicity 21%; major 14%. Reversible in 79% after dose reduction, interruption, HF therapy, or continued treatment with monitoring. Established HF not reported as a dedicated stratum.
Glen et al. [10] Retrospective longitudinal cohort; regional cancer network, United Kingdom; June 2017 to June 2020.	*n* = 63 patients with melanoma treated in routine care.	Combined BRAF and MEK inhibition (routine-care regimens).	Routine-care echocardiography during treatment; surveillance was not protocolized and analyzable data were absent in approximately half of patients by 24 weeks [13].	IC-OS definitions of mild, moderate and severe CTRCD; baseline risk assigned with the HFA-ICOS tool.	CTRCD 27% (mild 17%, moderate 10%); no severe CTRCD and no symptomatic HF; 82% of CTRCD occurred in patients classified as low or medium baseline risk. Pre-existing left ventricular dysfunction was rare and not analyzed separately.
Pedersen et al. [11] Retrospective real-world cohort; Eastern Denmark; 2019–2022.	*n* = 108 patients with metastatic melanoma.	Encorafenib + binimetinib only.	MUGA at baseline and every 3 months. Median time to any LVEF decline 90 days (134 days for major events); no significant new decline observed beyond 6 months of treatment.	Major cardiotoxicity: LVEF fall >10 pp to LVEF < 50%. Minor cardiotoxicity: LVEF fall >15 pp with LVEF remaining >50%. MUGA only; no strain or biomarker criterion.	Minor cardiotoxicity 18%, all asymptomatic; major cardiotoxicity 6%, of whom two required intervention. Single-regimen cohort; HF status not analyzed.
Glen et al. [13] Prospective observational cohort; regional cancer network, United Kingdom; 2021–2023.	*n* = 61 analyzed (63 recruited); performance status 0–1 and predicted survival ≥6 months required; 79% treated in the adjuvant setting; only 1 participant (1.6%) had pre-existing left ventricular systolic dysfunction.	Dabrafenib + trametinib (82%) or encorafenib + binimetinib (18%).	Protocolized assessment at baseline and at 4, 12 and 24 weeks: 7-day home blood pressure, echocardiography with GLS, ECG, high-sensitivity troponin T and NT-proBNP, plus a stress-perfusion cardiac magnetic resonance substudy (*n* = 20). 93.4% completed 24 weeks; follow-up limited to 6 months.	IC-OS CTRCD, including mild CTRCD defined by a >15% relative fall in GLS and/or a new biomarker rise with LVEF ≥ 50%; treatment-associated hypertension defined on 7-day average home readings.	CTRCD 45.9% (of these, 85.7% mild, 10.7% moderate, 3.6% severe); moderate or severe CTRCD in 6.6% of the cohort, all evident by 4 weeks and at least partially reversible; hypertension 45.9%; higher baseline NT-proBNP in those developing CTRCD (109 vs. 54 pg/mL; *p* = 0.047); no moderate or severe CTRCD in the low baseline-risk group. Essentially an HF-free population.
Barbieri et al. [12] Pharmacovigilance disproportionality analysis; FAERS; 2014–2023.	18,370 reports listing a BRAF or MEK inhibitor, of which 1591 (8.7%) reported a cardiovascular event.	All six agents, as monotherapy and as the three approved combinations.	Not applicable: spontaneous reports without protocolized follow-up, denominator, or baseline cardiac characterization.	Standardized MedDRA Query groupings for bradyarrhythmias, tachyarrhythmias, cardiac failure, cardiomyopathy, thrombotic events, ischemic heart disease and myocarditis/pericarditis; signals of disproportionate reporting.	Sixty-four clinically relevant signals, including QT prolongation, atrial fibrillation and disseminated intravascular coagulation with dabrafenib + trametinib, and cardiac failure and pulmonary embolism with vemurafenib + cobimetinib. Signal generation only; cannot estimate incidence or inform HF-specific risk.

**Table 6:** Management of established heart failure medication during systemic melanoma therapy. The default is continuation of guideline-directed medical therapy; the actions listed are situational adjustments rather than contraindications. ARNI, angiotensin receptor–neprilysin inhibitor; DOAC, direct oral anticoagulant; INR, international normalized ratio; LMWH, low-molecular-weight heparin; MRA, mineralocorticoid-receptor antagonist; P-gp, P-glycoprotein.

Drug Class or Intervention	Position During Melanoma Therapy	Specific Interaction or Interference	Practical Action
ACE inhibitors, ARBs, ARNI	Continue. Do not stop pre-emptively before BRAF/MEK inhibitors or ICIs.	No clinically important cytochrome interaction. Risk is pharmacodynamic: hypotension and acute kidney injury during diarrhea, vomiting, pyrexia or reduced intake. Sacubitril raises BNP but not NT-proBNP.	Temporarily reduce rather than withdraw during intercurrent illness and re-uptitrate early. Monitor creatinine, potassium and blood pressure at each cycle. Use NT-proBNP, never BNP, to track treatment-associated change in patients on an ARNI.
Mineralocorticoid-receptor antagonists	Continue when indicated for HFrEF.	Eplerenone is a sensitive CYP3A4 substrate; spironolactone is not. Hyperkalemia risk rises with acute kidney injury; high-dose corticosteroids for immune-related toxicity instead promote hypokalemia and QT risk.	Check potassium and renal function at every cycle and during any diarrheal illness. If a strong CYP3A4 inhibitor is unavoidable, substitute spironolactone or reduce the eplerenone dose rather than stopping mineralocorticoid blockade.
Beta-blockers	Continue at the established dose in HFrEF.	Additive bradycardia and conduction slowing with ICI-associated myocarditis and with QT-prolonging BRAF/MEK regimens. Bisoprolol is partly CYP3A4-dependent; carvedilol, metoprolol and nebivolol are CYP2D6 substrates and may be affected by enzyme induction.	Do not attribute new atrioventricular block or bradycardia during ICI therapy to the beta-blocker: investigate for myocarditis. Titrate to heart rate and blood pressure rather than to a nominal dose during induction.
SGLT2 inhibitors	Continue when indicated; not an established cardioprotective intervention against these specific toxicities.	No significant cytochrome interaction. Risk of euglycemic ketoacidosis with reduced intake, diarrhea, sepsis, fasting or surgery; genital infection risk increases under high-dose corticosteroids.	Give written sick-day rules and withhold temporarily during significant gastrointestinal toxicity, fasting, sepsis or the perioperative period. Monitor glucose and volume status when corticosteroids are added.
Loop diuretics	Continue; dose should be dynamic rather than fixed.	Hypokalemia and hypomagnesaemia amplify QT risk from encorafenib, vemurafenib and antiemetics. Combined diuresis and MEK inhibitor-associated diarrhea can precipitate hypovolemia and acute kidney injury.	Titrate to daily weight and congestion. Measure and replete potassium and magnesium before and during any QT-prolonging regimen; repeat with every episode of diarrhea or vomiting.
Digoxin	Continue only where clearly indicated; review the indication.	P-gp substrate with a narrow therapeutic index; exposure may rise with vemurafenib or encorafenib. Toxicity is potentiated by hypokalemia and by renal impairment.	Check serum digoxin concentration after starting or stopping the melanoma regimen and after any change in renal function. Maintain potassium in the mid-normal range.
Ivabradine	Review; often replaceable in this setting.	Sensitive CYP3A4 substrate with additional bradycardic and QT liability; exposure rises with CYP3A4 inhibitors and falls with the induction produced by dabrafenib or encorafenib.	Avoid with encorafenib-containing regimens. If retained, monitor heart rate and ECG closely and accept that efficacy may be reduced during enzyme induction.
Amiodarone and dronedarone	High-concern combinations; review the indication before starting therapy.	Additive QT prolongation and CYP3A4-mediated interaction, particularly with encorafenib.	Consider substitution or, where the antiarrhythmic is essential, prefer a BRAF/MEK pair with lower QT liability, correct electrolytes, and monitor ECG at baseline, at steady state and after any dose change.
Oral anticoagulants	Continue; anticoagulation is frequently required for atrial fibrillation or cancer-associated thrombosis.	Enzyme induction by dabrafenib or encorafenib reduces warfarin effect. Apixaban and rivaroxaban are CYP3A4 and P-gp substrates and exposure may fall; BRAF/MEK regimens themselves carry a thromboembolic signal [12].	Monitor INR closely and more frequently after any change in the melanoma regimen. For DOACs, consider LMWH when the interaction is unpredictable and thrombotic risk is high. Do not reduce anticoagulation intensity purely on interaction grounds.
Statins	Continue when indicated for concomitant atherosclerotic disease.	Simvastatin, lovastatin and atorvastatin depend on CYP3A4; rosuvastatin exposure may rise transiently with dabrafenib.	Prefer a less CYP3A-dependent statin such as pravastatin when a strong interaction is unavoidable; monitor for myalgia and check creatine kinase, remembering that creatine kinase elevation during ICI therapy may indicate myositis.
Cardiac implantable electronic devices	Continue; the device changes the monitoring plan rather than the treatment decision.	ICI myocarditis may present as high-grade block or ventricular arrhythmia; device diagnostics may be the earliest objective signal. Magnetic-resonance conditionality determines whether cardiac magnetic resonance is immediately available.	Interrogate at baseline, at any suspected myocarditis, and before cardiac magnetic resonance. Confirm and record magnetic-resonance-conditional status in advance, and review pacing dependence before starting checkpoint blockade.
High-dose corticosteroids for immune-related toxicity	Not a HF medicine, but a predictable HF stressor that must be planned for.	Sodium and water retention, hypertension, hyperglycemia and hypokalemia may precipitate congestion in HFrEF at the moment of lowest reserve.	Plan pre-emptive diuretic uptitration, daily weights, and daily potassium and glucose monitoring at the time corticosteroids are started [20]. Avoid infliximab in moderate-to-severe or decompensated HF; doses >5 mg/kg are contraindicated [46,47].

**Table 7:** Proposed baseline and follow-up cardiovascular assessment for patients with pre-existing heart failure receiving systemic melanoma therapy, presented separately for the two treatment classes. G, directly guideline-supported by the 2022 ESC cardio-oncology guidelines, the HFA-ICOS position statement, the IC-OS consensus definitions, heart failure guidelines, or approved product information [3,22,23,26,30,31,32,33,34,35]; AP, author-proposed risk-adapted extrapolation that is not stated in those sources and should not be cited as a guideline recommendation. CTRCD, cancer therapy-related cardiac dysfunction; G8, Geriatric 8 screening tool; HFA-ICOS, Heart Failure Association/International Cardio-Oncology Society; LVEF, left ventricular ejection fraction; NYHA, New York Heart Association; QTc, corrected QT interval.

Assessment	Baseline, Before First Exposure	Follow-Up During BRAF/MEK Inhibitor Therapy	Follow-Up During Immune Checkpoint Inhibitor Therapy	Basis
Symptoms and clinical examination	Habitual symptom pattern, NYHA class, functional capacity, dry weight, congestion status.	Every visit and at each dose modification; judge change from the documented baseline rather than absolute values.	Before every dose. New fatigue, dyspnea, chest pain, palpitations, myalgia, ptosis, diplopia, dysphagia or proximal weakness are possible myocarditis or myositis until excluded.	G
Blood pressure	Office measurement and, where feasible, a 7-day home series; confirm control before starting.	Home monitoring for 12 weeks, reviewed weekly in the first month; treat new or worsening hypertension promptly.	Every visit; here hypotension is the more informative direction.	G; AP for the home protocol
12-lead ECG	Rhythm, conduction, QTc and all pre-existing abnormalities recorded.	At 2–4 weeks, after any dose change, and whenever a QT-prolonging drug or electrolyte disturbance is added; mandatory with encorafenib or vemurafenib.	Baseline and any new symptom. New atrioventricular block, bundle branch block or ventricular arrhythmia is myocarditis until proven otherwise.	G; AP for the intervals
High-sensitivity cardiac troponin	Mandatory; establishes the chronically abnormal reference value.	Not routine; measure with new symptoms, ECG change or falling ventricular function.	Before doses 2, 3 and 4, then symptom-triggered. The signal is a rise above the personal baseline, not a population cut-point.	G at baseline; AP for serial testing
BNP or NT-proBNP	Mandatory. NT-proBNP only in patients receiving sacubitril/valsartan.	With clinical change, congestion, interruption or resumption; always against the personal baseline.	Baseline, and with suspected myocarditis or decompensation.	G
Echocardiography with LVEF and global longitudinal strain	LVEF, strain where feasible, diastolic indices, left atrial volume index, right ventricular function, pulmonary pressures and valves.	Approximately 4-monthly at high or very high risk. An additional study at approximately 4 weeks is proposed for unstable or very-high-risk patients [13]. Repeat after interruption and after recovery.	Not routine; urgent when myocarditis is suspected and repeated during immunosuppression. A normal LVEF does not exclude myocarditis.	G for the 4-monthly interval [3]; AP for the 4-week study
Weight, electrolytes and renal function	Weight, potassium, magnesium, sodium, creatinine and estimated glomerular filtration rate.	Potassium and magnesium before and during any QT-prolonging regimen and with every episode of diarrhea or vomiting; daily weight during monitored initiation.	Daily weight, potassium and glucose whenever high-dose corticosteroids are started.	G
Cardiac magnetic resonance and advanced evaluation	Not routine; consider if echocardiography is non-diagnostic or the phenotype is unexplained.	If dysfunction does not recover or the pattern is atypical [8].	Early when myocarditis is suspected; a negative study does not exclude it and must not delay corticosteroids. Endomyocardial biopsy if tests conflict or disease is refractory.	G
Device interrogation	All device-bearing patients; record pacing dependence and magnetic-resonance-conditional status.	With any arrhythmia, syncope or unexplained deterioration.	Baseline, any suspected myocarditis, and before cardiac magnetic resonance; may give the earliest objective signal.	AP
Multidisciplinary and geriatric input	Cardio-oncology review in every patient with established HF; G8 screening from 70 years of age.	Re-review at any re-grading, interruption, dose reduction or rechallenge.	The same, with immediate review at the first suspicion of myocarditis.	G for screening [28,29]; AP for the triggers

**Table 8:** Second-line and supportive strategies for severe or corticosteroid-refractory ICI-associated myocarditis.

Agent/Strategy	Evidence Base in ICI Myocarditis	Potential Role	HF-Specific Cautions
Abatacept ± ruxolitinib [45]	Mechanistic rationale and observational/case-series evidence; no randomized trial.	Severe or refractory disease in expert centers, particularly when rapid T-cell costimulation blockade is desired.	Monitor infection, cytopenias, hepatic effects, and hemodynamics. Dosing and pharmacodynamic targets remain non-standardized.
Mycophenolate mofetil [50,51]	Case reports/series and guideline-listed rescue option.	Adjunct for persistent myocardial inflammation or steroid-sparing treatment.	Delayed onset; infection and cytopenia risk. Not proven superior to other rescue strategies.
IVIG and/or plasma exchange [43,51]	Case-level evidence, especially in neuromuscular overlap.	Useful when myocarditis coexists with myositis/myasthenia, bulbar weakness, or respiratory compromise.	Volume load, thrombosis, renal dysfunction, and vascular-access considerations are relevant in HF.
Anti-thymocyte globulin or alemtuzumab [50,51]	Limited rescue reports.	Potential option for fulminant, refractory T-cell-mediated disease.	Profound immunosuppression, infusion reactions, infection, and cytopenias require intensive specialist care.
Tocilizumab [50]	Isolated reports/very small series.	Selected cytokine-driven refractory presentations.	Evidence is insufficient for routine use; monitor infection, liver tests, cytopenias, and gastrointestinal risk.
Infliximab [46,47]	Small retrospective case series; off-label and conflicting with HF safety concerns.	Not a broadly interchangeable rescue agent.	Generally avoid in moderate-to-severe or decompensated HF. Doses >5 mg/kg are contraindicated in moderate/severe HF; lower doses still require substantial caution.
Mechanical support and critical care	Supportive standard of care rather than immunosuppression.	Shock, malignant arrhythmia, high-grade block, or respiratory muscle failure.	Early ICU, electrophysiology, advanced HF, and neuromuscular involvement may be lifesaving while immunosuppression takes effect.

**Table 9:** Domain grading and decision output for the four illustrative composite cases described in Section 3.7. The cases are author-constructed teaching composites and do not represent identifiable patients.

Case	Domain 1: HF Substrate and Stability	Domain 2: Oncological Urgency	Domain 3: Toxicity Phenotype Match	Domain 4: Detectability and Priorities	Composite Pattern and Decision Output
Case 1 Stable HFrEF, BRAF V600E melanoma, no visceral crisis	Amber: LVEF 38%, NYHA II, stable 18 months on full guideline-directed therapy; reserve reduced but nothing unstable.	Amber: therapy clearly indicated; a 1–2 week window for baseline assessment is safe; an alternative class exists.	Red for BRAF/MEK inhibition (direct overlap with reduced LVEF); amber for ICIs (no prior myocarditis or conduction disease).	Green: interpretable baseline, prompt access to imaging and biomarkers, reliable attendance and support.	One red domain, regimen-specific. Output: modify the sequence—checkpoint blockade first with early ECG and troponin; BRAF/MEK inhibition retained as second line with a mandated 4-week echocardiogram.
Case 2 Recent decompensation, rapidly progressive melanoma	Red: HF admission 12 days earlier, unstable diuretic requirement, NT-proBNP falling but still markedly elevated.	Red: symptomatic liver metastases, rising LDH, performance status 2; no alternative with an acceptable time to response.	Red for the oncologically necessary class; within-class choice matters (lower QT liability and CYP3A4 dependence preferred with amiodarone).	Amber: monitoring achievable only in a supervised setting.	Three red domains including Domain 1. Output: initiate under enhanced monitoring after multidisciplinary review, with predefined escalation criteria and an explicit record of why delay was unsafe.
Case 3 Suspected ICI myocarditis on chronic HF	Red on re-grading: new conduction disease and a marked troponin rise from a chronically abnormal baseline, with unchanged LVEF.	Amber: disease controlled to date; a BRAF-directed alternative exists.	Red for continued checkpoint blockade; conduction and myositis overlap dominate the phenotype.	Green for detection, but reversibility is uncertain and corticosteroid therapy is itself a congestive stressor.	Output: immediate withholding, urgent multimodality assessment, methylprednisolone within 24 h, pre-emptive HF decongestion, infliximab excluded in advance; change in class rather than rechallenge.
Case 4 HFpEF, frailty, limited monitorability	Amber: preserved LVEF but congestion-prone, atrial fibrillation, chronic kidney disease stage 3b.	Amber: effective treatment indicated; BRAF wild-type; so, checkpoint blockade is the only option.	Amber to red: conduction disease and immune-mediated colitis would be poorly tolerated in a diuretic-dependent patient with renal impairment.	Red: distance, absent caregiver support, G8 score 11, patient priorities favoring independence.	One red domain (Domain 4). Output: restructure care—single-agent anti-PD-1, comprehensive geriatric assessment, shared-care monitoring, written sick-day rules and symptom-triggered pathway, documented goals of care.

**Table 10:** What the four-domain framework does and does not do. The table is provided so that the framework cannot be cited for a purpose it cannot serve. Every statement in the left-hand column is a procedural claim; none is an empirical claim about risk, safety or outcome.

Attribute	What the Framework Does	What the Framework Does Not Do
Derivation	Organizes four questions that clinicians already answer implicitly when treating a patient with pre-existing HF, chosen because they determine what happens to the patient.	It was not derived from a dataset, a systematic evidence synthesis, a modeling exercise, or a consensus process external to the authors.
Grading	Assigns ordinal green, amber and red categories against written descriptors so that two clinicians assess the same features.	It does not assign numerical weights, generate a score, or permit domains to be summed, averaged or traded off arithmetically.
Precedence rules	State which domain governs when two or more are abnormal, and make the reasoning behind that precedence explicit.	They were not tested against an alternative rule or against outcomes; they encode a judgement about which harm is less reversible.
Output	Produces one of five documented strategies, together with the reasoning, the trade-off accepted, and the criteria that would reverse it.	It does not produce a recommendation, a probability, an estimate of expected benefit, or authorization to treat or to withhold treatment.
Relation to guidelines	Operationalizes ESC and IC-OS principles for a situation those documents do not address, namely a cardiac abnormality that precedes therapy.	It does not replace, extend or override any guideline recommendation. Where the two diverge, the guideline prevails and the divergence is recorded.
Validation status	Is explicitly hypothesis-generating and is offered so that its components can be evaluated prospectively.	It has not been validated, calibrated, or compared with unstructured clinical judgement, and no dataset currently exists that would allow this.
Intended use	Structures a multidisciplinary discussion and the record that discussion produces so that decisions can be taught, audited and revised.	It is not a risk score, a triage instrument, a substitute for cardio-oncology review, or a basis for withholding effective therapy.
Citation	May be cited as a proposed decision structure for an understudied population.	It must not be cited as evidence, as a validated tool, or as a guideline-endorsed pathway.

**Table 11:** Patient-level priorities to be established and recorded in older patients with metastatic melanoma and pre-existing heart failure. These items belong to Domain 4 of the framework. They are author-proposed (AP): no validated instrument captures them in this population, and they are offered as a documentation standard rather than as a scored assessment.

Priority	The Question, Asked Explicitly	How the Answer Changes the Plan	What is Recorded
Overall survival relative to other goals	Is the aim the longest possible survival, or the most predictable course, even at some cost in expected survival?	Determines whether combination checkpoint blockade or an intensive sequence is pursued, or whether a single-agent, more predictable regimen is preferred.	The stated aim in the patient’s own words, and the alternative that was declined.
Quality of life	Which symptoms or limitations would the patient least tolerate, and what would make a treatment not worth continuing?	Identifies the toxicities that justify dose reduction or interruption before they reach a formal severity grade, and those the patient would accept.	The named symptoms and the threshold that would trigger a review.
Avoidance of hospitalization	How much does the patient value days at home, and has a previous admission changed that view?	Favors the regimen and setting least likely to generate admissions for this patient; strengthens the case for pre-emptive decongestion, sick-day rules and a direct contact route.	The weight given to this endpoint, and any prior admission that informed it.
Willingness to accept frequent monitoring	Can the patient attend weekly visits in the first month, and does he or she wish to?	Determines whether a monitoring-dependent strategy is deliverable at all; if it is not, the plan is restructured under the fourth precedence rule rather than the treatment withheld.	The agreed visit burden, the shared-care arrangement, and who performs each test.
Oral versus infusion treatment	Where both are oncologically reasonable, does the patient prefer daily tablets at home or intermittent infusions at the center?	Oral BRAF/MEK inhibition avoids travel but requires adherence, home blood-pressure monitoring and interaction discipline; infusion checkpoint blockade removes the daily burden but requires attendance and imposes travel.	The preference, the reason for it, and whether it was overridden on oncological grounds.
Functional expectation	What level of independence or mobility must be preserved for the patient to consider treatment worthwhile?	Provides a prospective threshold that can be applied if it is reached, rather than a judgement improvised during a crisis.	The functional threshold, stated concretely, and the date it was agreed.

## Data Availability

No new data were generated or analyzed in this study. Data sharing is not applicable. All information is derived from previously published studies, which are cited within this manuscript.

## Supplementary Materials

The following supporting information can be downloaded at: https://www.mdpi.com/article/10.3390/jcm15187022/s1, Table S1, Drug-specific pharmacokinetic and pharmacodynamic interaction framework for common BRAF/MEK inhibitor regimens; Table S2, Evidence basis of the principal recommendations in this review.

## Abbreviations

The following abbreviations are used in this manuscript:

anti-CTLA-4	Anti-cytotoxic T-lymphocyte-associated protein 4
anti-PD-1	Anti-programmed cell death protein 1
anti-PD-L1	Anti-programmed death-ligand 1
BNP	B-type natriuretic peptide
BRAF	B-Raf proto-oncogene, serine/threonine kinase
CMR	Cardiac magnetic resonance
CTRCD	Cancer therapy-related cardiac dysfunction
CYP	Cytochrome P450
DDI	Drug–drug interaction
Drug-PIN	Drug–drug interaction risk prediction score
ECG	Electrocardiography
EMB	Endomyocardial biopsy
ERK	Extracellular signal-regulated kinase
ESC	European Society of Cardiology
FAERS	FDA Adverse Event Reporting System
FDA	Food and Drug Administration
GDMT	Guideline-directed medical therapy
GLS	Global longitudinal strain
HF	Heart failure
HFA	Heart Failure Association
HFA-ICOS	Heart Failure Association/International Cardio-Oncology Society
HFrEF	Heart failure with reduced ejection fraction
ICI	Immune checkpoint inhibitor
ICOS/IC-OS	International Cardio-Oncology Society
IL-17A	Interleukin-17A
INR	International normalized ratio
IVIG	Intravenous immunoglobulin
LVEF	Left ventricular ejection fraction
MAPK	Mitogen-activated protein kinase
MDT	Multidisciplinary team
MEK	Mitogen-activated protein kinase kinase
MMF	Mycophenolate mofetil
MUGA	Multigated acquisition scan
NT-proBNP	N-terminal pro-B-type natriuretic peptide
NYHA	New York Heart Association
PD-1	Programmed cell death protein 1
PD-L1	Programmed death-ligand 1
QTc	Corrected QT interval
RAAS	Renin–angiotensin–aldosterone system
ARNI	Angiotensin receptor–neprilysin inhibitor
ATG	Anti-thymocyte globulin
CK	Creatine kinase
cTnI	Cardiac troponin I
cTnT	Cardiac troponin T
TTE	Transthoracic echocardiography
ACEi	Angiotensin-converting enzyme inhibitor
AF	Atrial fibrillation
AKI	Acute kidney injury
ARB	Angiotensin receptor blocker
AV	Atrioventricular
CKD	Chronic kidney disease
DOAC	Direct oral anticoagulant
eGFR	Estimated glomerular filtration rate
G8	Geriatric 8 screening tool
HFimpEF	Heart failure with improved ejection fraction
HFmrEF	Heart failure with mildly reduced ejection fraction
HFpEF	Heart failure with preserved ejection fraction
LDH	Lactate dehydrogenase
LMWH	Low-molecular-weight heparin
LV	Left ventricular
MRA	Mineralocorticoid-receptor antagonist
P-gp	P-glycoprotein
RV	Right ventricular
SGLT2	Sodium–glucose cotransporter 2
SMQ	Standardized MedDRA Query
VTE	Venous thromboembolism
G	Guideline-supported statement (defined in Section 2.5)
AP	Author-proposed statement (defined in Section 2.5)

## Appendix A. Reproducible Search Strategy

MEDLINE/PubMed: ((melanoma[Title/Abstract] OR “cutaneous melanoma”[Title/Abstract]) AND ((“BRAF inhibitor”[Title/Abstract] OR “MEK inhibitor”[Title/Abstract] OR dabrafenib[Title/Abstract] OR trametinib[Title/Abstract] OR encorafenib[Title/Abstract] OR binimetinib[Title/Abstract] OR vemurafenib[Title/Abstract] OR cobimetinib[Title/Abstract] OR “immune checkpoint inhibitor”[Title/Abstract] OR nivolumab[Title/Abstract] OR pembrolizumab[Title/Abstract] OR ipilimumab[Title/Abstract]) AND (“heart failure”[Title/Abstract] OR cardiotoxic*[Title/Abstract] OR myocarditis[Title/Abstract] OR “left ventricular dysfunction”[Title/Abstract] OR CTRCD[Title/Abstract] OR “cardiac dysfunction”[Title/Abstract] OR “cardiovascular toxicity”[Title/Abstract] OR cardio-oncology[Title/Abstract]))) with publication-date limits from 1 January 2015 to 30 April 2026; targeted updates were performed through 31 July 2026.

Embase: (‘melanoma’/exp OR melanoma:ti,ab OR ‘cutaneous melanoma’:ti,ab) AND ((‘braf inhibitor’/exp OR ‘mek inhibitor’/exp OR dabrafenib:ti,ab OR trametinib:ti,ab OR encorafenib:ti,ab OR binimetinib:ti,ab OR vemurafenib:ti,ab OR cobimetinib:ti,ab OR ‘immune checkpoint inhibitor’/exp OR nivolumab:ti,ab OR pembrolizumab:ti,ab OR ipilimumab:ti,ab) AND (‘heart failure’/exp OR cardiotoxic*:ti,ab OR myocarditis:ti,ab OR ‘left ventricular dysfunction’:ti,ab OR CTRCD:ti,ab OR ‘cardiac dysfunction’:ti,ab OR ‘cardiovascular toxicity’:ti,ab OR ‘cardio oncology’:ti,ab)) AND [english]/lim AND [2015–2026]/py.

Cochrane Library: (melanoma):ti,ab,kw AND (BRAF OR MEK OR dabrafenib OR trametinib OR encorafenib OR binimetinib OR vemurafenib OR cobimetinib OR “immune checkpoint inhibitor” OR nivolumab OR pembrolizumab OR ipilimumab):ti,ab,kw AND (“heart failure” OR cardiotoxicity OR myocarditis OR “left ventricular dysfunction” OR CTRCD OR “cardiac dysfunction” OR “cardiovascular toxicity” OR cardio-oncology):ti,ab,kw. Citation searching and targeted searches were additionally used for official product information, drug–drug interactions, ICI-myocarditis management, and geriatric oncology.

## References

[1] Robert C., Grob J.J., Stroyakovskiy D., Karaszewska B., Hauschild A., Levchenko E., Chiarion-Sileni V., Schachter J., Garbe C., Bondarenko I. (2019). Five-Year Outcomes with Dabrafenib plus Trametinib in Metastatic Melanoma. N. Engl. J. Med..

[2] Wolchok J.D., Chiarion-Sileni V., Rutkowski P., Cowey C.L., Schadendorf D., Wagstaff J., Queirolo P., Dummer R., Butler M.O., Hill A.G. (2025). Final, 10-Year Outcomes with Nivolumab plus Ipilimumab in Advanced Melanoma. N. Engl. J. Med..

[3] Lyon A.R., López-Fernández T., Couch L.S., Asteggiano R., Aznar M.C., Bergler-Klein J., Boriani G., Cardinale D., Cordoba R., Cosyns B. (2022). 2022 ESC Guidelines on Cardio-Oncology Developed in Collaboration with the European Hematology Association, the European Society for Therapeutic Radiology and Oncology and the International Cardio-Oncology Society. Eur. Heart J..

[4] Howell A.V., Gebregziabher M., Thiers B.H., Graboyes E.M., Paulos C.M., Wrangle J.M., Hunt K.J., Wallace K. (2022). Association of Age with Survival in Older Patients with Cutaneous Melanoma Treated with Immune Checkpoint Inhibitors. J. Geriatr. Oncol..

[5] Baethge C., Goldbeck-Wood S., Mertens S. (2019). SANRA—A Scale for the Quality Assessment of Narrative Review Articles. Res. Integr. Peer Rev..

[6] Page M.J., McKenzie J.E., Bossuyt P.M., Boutron I., Hoffmann T.C., Mulrow C.D., Shamseer L., Tetzlaff J.M., Akl E.A., Brennan S.E. (2021). The PRISMA 2020 Statement: An Updated Guideline for Reporting Systematic Reviews. BMJ.

[7] Nishizawa A., Kawakami M., Kitahara Y. (2024). Case Report: A Case of Metastatic BRAF V600-Mutated Melanoma with Heart Failure Treated with Immune Checkpoint Inhibitors and BRAF/MEK Inhibitors. Front. Oncol..

[8] Benko J., Péč M.J., Tomčová Z., Péčová M., Samoš M. (2024). Cardiotoxicity of BRAF/MEK Inhibitors Mimicking Apical Hypertrophic Cardiomyopathy. Cureus.

[9] Oddershede J.K., Meklenborg I.K., Bastholt L., Guldbrandt L.M., Schmidt H., Friis R.B. (2025). Cardiotoxicity in Patients with Metastatic Melanoma Treated with BRAF/MEK Inhibitors: A Real-World Analysis of Incidence, Risk Factors, and Reversibility. Acta Oncol..

[10] Glen C., Adam S., McDowell K., Waterston A., Tan Y.Y., Petrie M.C., Coats C.J., Lang N.N. (2023). Cardiotoxicity of BRAF/MEK Inhibitors: A Longitudinal Study Incorporating Contemporary Definitions and Risk Scores. JACC CardioOncology.

[11] Pedersen S., Nielsen M.Ø., Donia M., Svane I.M., Zerahn B., Ellebaek E. (2024). Real-World Cardiotoxicity in Metastatic Melanoma Patients Treated with Encorafenib and Binimetinib. Cancers.

[12] Barbieri M.A., Russo G., Cicala G., Zito C., Spina E., Silvestris N., Santarpia M. (2025). Cardiovascular Safety Profile of BRAF and MEK Inhibitors in Melanoma: FAERS Data through a Retrospective Disproportionality Analysis, 2014–2023. Cancers.

[13] Glen C., Dobbin S.J.H., Mangion K., Henderson A., Brooksbank K., Coats C.J., Epstein F.H., Kellman P., Butler E., Evans T.R.J. (2025). Prospective Evaluation of the Cardiovascular Effects of BRAF and MEK Inhibitors in Patients with Melanoma. JACC CardioOncology.

[14] Heinzerling L., Ott P.A., Hodi F.S., Husain A.N., Tajmir-Riahi A., Tawbi H., Pauschinger M., Gajewski T.F., Lipson E.J., Luke J.J. (2016). Cardiotoxicity Associated with CTLA4 and PD1 Blocking Immunotherapy. J. Immunother. Cancer.

[15] Ostos-Mendoza K.C., Saraiba-Rabanales V., Gutierrez-Gallegos P., Wang E., Koutroumpakis E., Kumar S., Khalaf S., Ali H.J., Deswal A., Palaskas N.L. (2025). Immune Checkpoint Inhibitor-Associated Myocarditis: A Review of Current State of Knowledge. J. Cardiovasc. Aging.

[16] Allenbach Y., Anquetil C., Manouchehri A., Benveniste O., Lambotte O., Lebrun-Vignes B., Spano J.P., Ederhy S., Klatzmann D., Rosenzwajg M. (2020). Immune Checkpoint Inhibitor-Induced Myositis, the Earliest and Most Lethal Complication among Rheumatic and Musculoskeletal Toxicities. Autoimmun. Rev..

[17] Wang D.Y., Salem J.E., Cohen J.V., Chandra S., Menzer C., Ye F., Zhao S., Das S., Beckermann K.E., Ha L. (2018). Fatal Toxic Effects Associated with Immune Checkpoint Inhibitors: A Systematic Review and Meta-Analysis. JAMA Oncol..

[18] Mahmood S.S., Fradley M.G., Cohen J.V., Nohria A., Reynolds K.L., Heinzerling L.M., Sullivan R.J., Damrongwatanasuk R., Chen C.L., Gupta D. (2018). Myocarditis in Patients Treated with Immune Checkpoint Inhibitors. J. Am. Coll. Cardiol..

[19] Wang C., Zhao G., Zhang Z., Yang L., Liu S., Li G., Wang H., Huang J., Wang S., Li N. (2023). Immune Checkpoint Inhibitor-Associated Myocarditis: A Systematic Analysis of Case Reports. Front. Immunol..

[20] Palaskas N.L., Siddiqui B.A., Deswal A. (2024). Steroids in Immune Checkpoint Inhibitor Myocarditis. JACC CardioOncology.

[21] Salem J.E., Manouchehri A., Moey M., Lebrun-Vignes B., Bastarache L., Pariente A., Gobert A., Spano J.P., Balko J.M., Bonaca M.P. (2018). Cardiovascular Toxicities Associated with Immune Checkpoint Inhibitors: An Observational, Retrospective, Pharmacovigilance Study. Lancet Oncol..

[22] Herrmann J., Lenihan D., Armenian S., Barac A., Blaes A., Burns K., Cardinale D., Carver J., Dent S., Ky B. (2022). Defining Cardiovascular Toxicities of Cancer Therapies: An International Cardio-Oncology Society Consensus Statement. Eur. Heart J..

[23] McDonagh T.A., Metra M., Adamo M., Gardner R.S., Baumbach A., Böhm M., Burri H., Butler J., Čelutkienė J., Chioncel O. (2021). 2021 ESC Guidelines for the Diagnosis and Treatment of Acute and Chronic Heart Failure. Eur. Heart J..

[24] Anker S.D., Butler J., Filippatos G., Ferreira J.P., Bocchi E., Böhm M., Brunner-La Rocca H.P., Choi D.J., Chopra V., Chuquiure-Valenzuela E. (2021). Empagliflozin in Heart Failure with a Preserved Ejection Fraction. N. Engl. J. Med..

[25] Packer M., Anker S.D., Butler J., Filippatos G., Pocock S.J., Carson P., Januzzi J., Verma S., Tsutsui H., Brueckmann M. (2020). Cardiovascular and Renal Outcomes with Empagliflozin in Heart Failure. N. Engl. J. Med..

[26] Lyon A.R., Dent S., Stanway S., Earl H., Brezden-Masley C., Cohen-Solal A., Tocchetti C.G., Moslehi J., Groarke J.D., Bergler-Klein J. (2020). Baseline Cardiovascular Risk Assessment in Cancer Patients Scheduled to Receive Cardiotoxic Cancer Therapies: A Position Statement and New Risk Assessment Tools from the Heart Failure Association in Collaboration with the International Cardio-Oncology Society. Eur. J. Heart Fail..

[27] Stoff R., Markovic S.N., McWilliams R.R., Kottschade L.A., Montane H.N., Dimou A., Dudek A.Z., Tan W., Dronca R.S., Seetharam M. (2024). Real-World Evidence on Efficacy and Toxicity of Targeted Therapy in Older Melanoma Patients Treated in a Tertiary-Hospital Setting. Melanoma Res..

[28] Tran Van Hoi E., Trompet S., Van Holstein Y., Van Den Bos F., Van Heemst D., Codrington H., Labots G., Lohman S., Ozkan A., Portielje J. (2024). Toxicity in Older Patients with Cancer Receiving Immunotherapy: An Observational Study. Drugs Aging.

[29] Mac Eochagain C., Neuendorff N.R., Gente K., Leipe J., Verhaert M., Sam C., de Glas N., Kadambi S., Canin B., Gomes F. (2025). Management of Immune Checkpoint Inhibitor-Associated Toxicities in Older Adults with Cancer: Recommendations from the International Society of Geriatric Oncology (SIOG). Lancet Oncol..

[30] National Library of Medicine DailyMed: TAFINLAR (Dabrafenib)—Full Prescribing Information. https://dailymed.nlm.nih.gov/dailymed/drugInfo.cfm?setid=fee1e6b1-e1a5-4254-9f2e-a70e0f8dbdea.

[31] National Library of Medicine DailyMed: MEKINIST (Trametinib)—Full Prescribing Information. https://dailymed.nlm.nih.gov/dailymed/drugInfo.cfm?setid=0002ad27-779d-42ab-83b5-bc65453412a1.

[32] National Library of Medicine DailyMed: BRAFTOVI (Encorafenib)—Full Prescribing Information. https://dailymed.nlm.nih.gov/dailymed/drugInfo.cfm?setid=235dfc38-0f0b-4037-b501-7a9f4294740c.

[33] National Library of Medicine DailyMed: MEKTOVI (Binimetinib)—Full Prescribing Information. https://dailymed.nlm.nih.gov/dailymed/drugInfo.cfm?setid=6c3408ac-d401-4925-8a03-26591afbc240.

[34] National Library of Medicine DailyMed: ZELBORAF (Vemurafenib)—Full Prescribing Information. https://dailymed.nlm.nih.gov/dailymed/drugInfo.cfm?setid=38eea320-7e0c-485a-bc30-98c3c45e2763.

[35] National Library of Medicine DailyMed: COTELLIC (Cobimetinib)—Full Prescribing Information. https://dailymed.nlm.nih.gov/dailymed/drugInfo.cfm?setid=c387579e-cee0-4334-bd1e-73f93ac1bde6.

[36] Glen C., Tan Y.Y., Waterston A., Evans T.R.J., Jones R.J., Petrie M.C., Lang N.N. (2022). Mechanistic and Clinical Overview Cardiovascular Toxicity of BRAF and MEK Inhibitors: JACC: CardioOncology State-of-the-Art Review. JACC CardioOncology.

[37] Atkins M.B., Lee S.J., Chmielowski B., Tarhini A.A., Cohen G.I., Truong T.G., Moon H.H., Davar D., O’Rourke M., Stephenson J.J. (2023). Combination Dabrafenib and Trametinib versus Combination Nivolumab and Ipilimumab for Patients with Advanced BRAF-Mutant Melanoma: The DREAMseq Trial—ECOG-ACRIN EA6134. J. Clin. Oncol..

[38] Mezi S., Botticelli A., Pomati G., Fiscon G., Verkhovskaia S., Amirhassankhani S., Pisegna S., Gentile G., Simmaco M., Gohlke B. (2025). Impact of Drug–Drug Interactions on Clinical Outcomes in Metastatic Melanoma Patients Treated with Combined BRAF/MEK Inhibitors: A Real-World Study. Pigment Cell Melanoma Res..

[39] Mezi S., Botticelli A., Scagnoli S., Pomati G., Fiscon G., De Galitiis F., Di Pietro F.R., Verkhovskaia S., Amirhassankhani S., Pisegna S. (2023). The Impact of Drug–Drug Interactions on the Toxicity Profile of Combined Treatment with BRAF and MEK Inhibitors in Patients with BRAF-Mutated Metastatic Melanoma. Cancers.

[40] University of Liverpool and Radboud University Medical Center Cancer Drug Interactions. https://cancer-druginteractions.org/.

[41] Zhang L., Awadalla M., Mahmood S.S., Nohria A., Hassan M.Z.O., Thuny F., Zlotoff D.A., Murphy S.P., Stone J.R., Golden D.L.A. (2020). Cardiovascular Magnetic Resonance in Immune Checkpoint Inhibitor-Associated Myocarditis. Eur. Heart J..

[42] Deharo F., Thuny F., Cadour F., Resseguier N., Meilhac A., Gaubert M., Dolladille C., Paganelli F., Alexandre J., Cautela J. (2023). Diagnostic Value of the International Society of Cardio-Oncology Definition for Suspected Immune Checkpoint Inhibitor-Associated Myocarditis. J. Am. Heart Assoc..

[43] Pathak R., Katel A., Massarelli E., Villaflor V.M., Sun V., Salgia R. (2021). Immune Checkpoint Inhibitor-Induced Myocarditis with Myositis/Myasthenia Gravis Overlap Syndrome: A Systematic Review of Cases. Oncologist.

[44] Rossi V.A., Gawinecka J., Dimitriou F., von Eckardstein A., Dummer R., Ruschitzka F., Matter C.M. (2023). Value of Troponin T versus I in the Diagnosis of Immune Checkpoint Inhibitor-Related Myocarditis and Myositis: Rechallenge?. ESC Heart Fail..

[45] Salem J.E., Bretagne M., Abbar B., Leonard-Louis S., Ederhy S., Redheuil A., Boussouar S., Nguyen L.S., Procureur A., Stein F. (2023). Abatacept/Ruxolitinib and Screening for Concomitant Respiratory Muscle Failure to Mitigate Fatality of Immune-Checkpoint Inhibitor Myocarditis. Cancer Discov..

[46] Zhang R.S., Padegimas A., Murphy K.M., Evans P.T., Peters C.J., Domenico C.M., Vidula M.K., Mather P.J., Cevasco M., Cohen R.B. (2021). Treatment of Corticosteroid-Refractory Immune Checkpoint Inhibitor Myocarditis with Infliximab: A Case Series. Cardio-Oncology.

[47] U.S. Food and Drug Administration REMICADE (Infliximab)—Full Prescribing Information. https://www.accessdata.fda.gov/drugsatfda_docs/label/2021/103772s5401lbl.pdf.

[48] Thavendiranathan P., Negishi T., Somerset E., Negishi K., Penicka M., Lemieux J., Aakhus S., Miyazaki S., Shirazi M., Galderisi M. (2021). Strain-Guided Management of Potentially Cardiotoxic Cancer Therapy. J. Am. Coll. Cardiol..

[49] Zhang L., Zlotoff D.A., Awadalla M., Mahmood S.S., Nohria A., Hassan M.Z.O., Thuny F., Zubiri L., Chen C.L., Sullivan R.J. (2020). Major Adverse Cardiovascular Events and the Timing and Dose of Corticosteroids in Immune Checkpoint Inhibitor-Associated Myocarditis. Circulation.

[50] Cautela J., Zeriouh S., Gaubert M., Bonello L., Laine M., Pinto J., Paganelli F., Thuny F. (2020). Intensified Immunosuppressive Therapy in Patients with Immune Checkpoint Inhibitor-Induced Myocarditis. J. Immunother. Cancer.

[51] Brahmer J.R., Abu-Sbeih H., Ascierto P.A., Brufsky J., Cappelli L.C., Cortazar F.B., Gerber D.E., Hamad L., Hansen E., Johnson D.B. (2021). Society for Immunotherapy of Cancer Clinical Practice Guideline on Immune Checkpoint Inhibitor-Related Adverse Events. J. Immunother. Cancer.

[52] Manta E., Iliakis P., Fragoulis C., Leontsinis I., Stamoulopoulos I., Chrysohoou C., Tsioufis K. (2025). Tracking Pathways Linking Obesity with Heart Failure. Nutrients.

