# Supplementary material for: Systemic Melanoma Therapy in Patients with Pre-Existing Heart Failure: An HF-Centered Decision Framework and Critical Narrative Review
Source: J Clin Med. 2026 Sep 10;15(18):7022. doi: 10.3390/jcm15187022 (PMC13607582; doi:10.3390/jcm15187022)
Supplement: Supplementary file 1 [file jcm-15-07022-s001.zip › jcm-4510570-supplementary.pdf]

Supplementary Materials

Table S1. Drug-specific pharmacokinetic and pharmacodynamic interaction framework for common BRAF/MEK inhibitor regimens.

Regimen/pathway	Interaction direction	Practical category	Cardiovascular interpretation
Dabrafenib + trametinib [30,31]	Strong CYP3A4 or CYP2C8 inhibitors increase dabrafenib exposure; strong CYP3A4 inducers reduce exposure. Dabrafenib induces CYP3A4/CYP2C9 and may alter transporter substrates.	Prefer alternatives to strong inhibitors/inducers; if unavoidable, follow label and monitor toxicity/efficacy. Monitor INR with warfarin.	Amlodipine/nifedipine and CYP3A-dependent statins are not uniformly contraindicated; induction may reduce efficacy. Rosuvastatin peak exposure may increase. Acid-reducing therapy is not a major dabrafenib interaction.
Encorafenib + binimetinib [32,33]	Strong/moderate CYP3A4 inhibitors increase encorafenib; strong CYP3A4 inducers reduce it. Encorafenib induces CYP3A4 and can affect OATP/BCRP substrates. Encorafenib prolongs QT.	Avoid strong/moderate CYP3A4 inhibitors when possible; reduce encorafenib dose if unavoidable. Avoid strong inducers and QT-prolonging combinations.	Amiodarone/dronedarone are high-concern combinations because of QT and CYP effects. Correct K+/Mg2+ and monitor ECG when any QT risk is present.
Vemurafenib + cobimetinib [34,35]	Strong CYP3A inhibitors/inducers can materially change exposure, especially cobimetinib. Vemurafenib inhibits CYP1A2 and has QT-prolonging potential.	Avoid strong CYP3A modifiers; follow cobimetinib dose instructions for unavoidable short-term moderate inhibitors. Avoid or closely manage additive QT risk.	Review antiarrhythmics, psychotropics, antiemetics, and electrolyte-depleting diuretics. Do not generalize one pair’s interaction to all BRAF/MEK regimens.

Regimen/pathway	Interaction direction	Practical category	Cardiovascular interpretation
All regimens [30–35,40]	Interaction significance depends on exact agent, dose, direction of exposure change, and therapeutic index.	Classify each pair as avoid/contraindicated, dose-adjust, monitor closely, or no important interaction expected.	Use the product label and a recognized oncology interaction checker; repeat review whenever therapy or concomitant medication changes.

Table S2. Evidence basis of the principal recommendations in this review. G, directly guideline-supported; AP, author-proposed risk-adapted synthesis. The table is intended to be used when the review is cited: statements labelled AP are the authors' proposals for an understudied population and carry no guideline authority.

Recommendation or component	Basis	What the cited source states	What this review adds
Baseline cardiovascular assessment before systemic therapy	G	ESC 2022 recommends baseline assessment and therapy-specific risk stratification before potentially cardiotoxic treatment [3,26].	Specifies the additional parameters required when the baseline is already abnormal, and the recording of personal reference values.
HFA-ICOS risk stratification, with previous HF or cardiomyopathy as a very-high-risk item	G	Stated in the HFA-ICOS position statement for the proforma entitled "RAF/MEK inhibitors" [26].	Notes that the item is ungraded by phenotype, stability or time since decompensation and generates no management pathway; the ordinal grading proposed instead (Table 2) is author-proposed.
Echocardiographic surveillance approximately every 4 months at high or very high risk	G	ESC 2022 cardio-oncology guidelines [3].	Retained unchanged as the default interval.
Additional reassessment at approximately 4 weeks in unstable or very-high-risk patients	AP	No guideline states this; it is derived from one prospective cohort of 61 analysed patients, essentially free of pre-existing HF, in which every moderate or severe CTRCD was evident at 4 weeks [13].	Offered as a defensible risk-adapted option, not a standard of care; imaging at 3 months instead does not deviate from guidance.
Four-domain grading, descriptors, precedence rules and composite patterns (Tables 2 and 3; Figures 1 and 2)	AP	No source proposes an HF-centred decision structure for this population.	Entirely author-derived; not weighted from outcome data, not validated, and not to be used or cited as a risk score.

Recommendation or component	Basis	What the cited source states	What this review adds
Phenotype-specific readiness and monitoring, including HFpEF, right ventricular failure, devices and advanced HF (Table 4; Section 3.1.4)	AP	Current guidance addresses cancer therapy-related dysfunction in a structurally normal heart and gives no phenotype-specific pathway here.	Author-derived proposals, constructed to be consistent with general HF guidance [23–25] but untested against melanoma-specific outcomes.
Continuation of guideline-directed HF therapy throughout melanoma treatment	G	HF guidelines support continuation, and no product label requires routine discontinuation of any HF drug class [23–25,30–35].	Adds the situational adjustments of Table 6, which mix label-derived statements with pharmacologically reasoned proposals such as avoiding ivabradine with encorafenib (author-proposed).
Use of NT-proBNP rather than BNP in patients receiving sacubitril/valsartan	G	Established pharmacology of neprilysin inhibition, reflected in HF guidance [23].	Applies the principle to cardio-oncology surveillance, where the error would otherwise be attributed to cancer therapy.
Immediate withholding of the checkpoint inhibitor and methylprednisolone 500–1000 mg/day within 24 h for suspected myocarditis	G	ESC 2022, cardio-oncology consensus, and observational data on corticosteroid timing [3,20,22,49].	Adds the HF-specific corollary that pre-emptive decongestion and daily electrolyte and glucose monitoring are planned at the same time (author-proposed).
Avoidance of infliximab in moderate-to-severe HF, with doses above 5 mg/kg contraindicated	G	Approved product information and HF safety data [46,47].	Applies the restriction prospectively to the written escalation plan, so the alternative is identified before the patient becomes refractory.
Interruption, resumption and rechallenge criteria referenced to the personal baseline	AP	IC-OS defines severity categories; it does not define interruption thresholds or management [22].	Author-derived, and requires the event first to be attributed among progression of the underlying HF, treatment-associated dysfunction and myocarditis.
Preference for an immunotherapy-first sequence in BRAF-mutant melanoma	G	DREAMseq showed an overall survival advantage for immunotherapy-first sequencing in eligible patients [37].	Guideline-supported at population level only. The caveat that it does not transfer automatically to unstable HF, prior myocarditis, severe conduction disease or a need for rapid response is author-proposed.

Recommendation or component	Basis	What the cited source states	What this review adds
SGLT2 inhibitors during melanoma therapy	G	Randomized HF trials across the ejection-fraction spectrum [24,25].	Guideline-supported only when independently indicated for HF. There is no evidence of class-specific protection against BRAF/MEK-associated CTRCD; that remains a hypothesis (author-proposed).
